# Shotgun Metagenomics Study Suggests Alteration in Sulfur Metabolism and Oxidative Stress in Children with Autism and Improvement after Microbiota Transfer Therapy

**DOI:** 10.3390/ijms232113481

**Published:** 2022-11-03

**Authors:** Khemlal Nirmalkar, Fatir Qureshi, Dae-Wook Kang, Juergen Hahn, James B. Adams, Rosa Krajmalnik-Brown

**Affiliations:** 1 Biodesign Center for Health Through Microbiomes, Arizona State University, Tempe, AZ 85287, USA; 2 Department of Biomedical Engineering, Rensselaer Polytechnic Institute, Troy, NY 12180, USA; 3 Center for Biotechnology and Interdisciplinary Studies, Rensselaer Polytechnic Institute, Troy, NY 12180, USA; 4 Department of Chemical and Biological Engineering, Rensselaer Polytechnic Institute, Troy, NY 12180, USA; 5 School for Engineering of Matter, Transport, and Energy, Arizona State University, Tempe, AZ 85287, USA; 6 School of Sustainable Engineering and the Built Environment, Arizona State University, Tempe, AZ 85281, USA

**Keywords:** autism spectrum disorder (ASD), gut microbiome, metagenomics, fecal microbiota transplant (FMT), microbiota transfer therapy (MTT)

## Abstract

Links between gut microbiota and autism spectrum disorder (ASD) have been explored in many studies using 16S rRNA gene amplicon and shotgun sequencing. Based on these links, microbiome therapies have been proposed to improve gastrointestinal (GI) and ASD symptoms in ASD individuals. Previously, our open-label microbiota transfer therapy (MTT) study provided insight into the changes in the gut microbial community of children with ASD after MTT and showed significant and long-term improvement in ASD and GI symptoms. Using samples from the same study, the objective of this work was to perform a deeper taxonomic and functional analysis applying shotgun metagenomic sequencing. Taxonomic analyses revealed that ASD Baseline had many bacteria at lower relative abundances, and their abundance increased after MTT. The relative abundance of fiber consuming and beneficial microbes including *Prevotella* (*P. dentalis*, *P. enoeca*, *P. oris*, *P. meloninogenica*), *Bifidobacterium bifidum*, and a sulfur reducer *Desulfovibrio piger* increased after MTT-10wks in children with ASD compared to Baseline (consistent at genus level with the previous 16S rRNA gene study). Metabolic pathway analysis at Baseline compared to typically developing (TD) children found an altered abundance of many functional genes but, after MTT, they became similar to TD or donors. Important functional genes that changed included: genes encoding enzymes involved in folate biosynthesis, sulfur metabolism and oxidative stress. These results show that MTT treatment not only changed the relative abundance of important genes involved in metabolic pathways, but also seemed to bring them to a similar level to the TD controls. However, at a two-year follow-up, the microbiota and microbial genes shifted into a new state, distinct from their levels at Baseline and distinct from the TD group. Our current findings suggest that microbes from MTT lead to initial improvement in the metabolic profile of children with ASD, and major additional changes at two years post-treatment. In the future, larger cohort studies, mechanistic in vitro experiments and metatranscriptomics studies are recommended to better understand the role of these specific microbes, functional gene expression, and metabolites relevant to ASD.

## 1. Introduction

Autism spectrum disorder (ASD) is a neurodevelopmental condition characterized by difficulties with social communication and restricted and repetitive behaviors [1]. Recently, the CDC reported that ASD is estimated to affect about 1 in 44 children in the United States [2]. ASD is commonly associated with the co-occurrence of several medical comorbidities including gastrointestinal (GI) disorders such as chronic constipation, diarrhea and abdominal pain [3,4,5,6]. In our previous study [7], we found that these GI problems began in infancy in all 18 children with ASD and had continued until treatment with MTT at ages 7–16 years. The gut microbiota of individuals with ASD, with and without the presence of co-occurring GI symptoms, has usually been reported to be distinct from their typically developing (TD) peers [7,8,9,10].

Previously, in our open-label study of microbiota transfer therapy (MTT) for children with ASD, MTT reduced GI symptoms severity by ~80% and ASD symptoms ~24% by the end of treatment [7]. Furthermore, the relative abundance of *Bifidobacterium*, *Prevotella*, and *Desulfovibrio* at genera level increased after 10 weeks of treatment [7]. A 2-year follow-up study after MTT of these same children showed a ~59% reduction in GI symptoms and a ~47% reduction in ASD symptoms compared with Baseline, and the abundance of *Bifidobacterium*, *Prevotella* and *Desulfovibrio* remained higher than Baseline [11]. Improvement in behavioral symptoms suggests that MTT may also help to restore the altered gut–brain connection [12].

The gut–brain axis is bidirectional; gut microbiota, metabolic function and metabolites play an important role in establishing GI and central nervous system (CNS) connections [13,14]. Microbial functional gene analysis from some limited cross-sectional studies in children with ASD showed an altered or depleted abundance of genes that encode for neurotransmitters [15] such as GABA [16], dopamine, serotonin, glutamate [17,18], and microbial detoxification [19].

Microbiome knowledge at the species/strain level of the metabolic pathways/genes present in the gut microbiota of ASD patients is limited [15,16,17,18,19], and this area has not been explored longitudinally using MTT treatment in children with autism. These limitations warrant an extensive study to understand the functional impact of gut microbiota before and after MTT.

Our previous open-label study of MTT involved 16S rRNA gene amplicon sequencing [7,11] and metabolomic [20] evaluations. Those studies revealed changes in microbial (genus level) and metabolomic profiles and an improvement in ASD and GI symptoms after MTT (10 wk and 2 yrs). The objective of this work was to perform shotgun metagenomic sequencing on these MTT samples. We hypothesized that shotgun metagenomics would provide more details on microbial taxonomy and the possible important genes/pathways changing with treatment.

## 2. Results

### 2.1. Many Bacterial Species Shifted after MTT, Mostly after 2 Years

**Figure 1:**
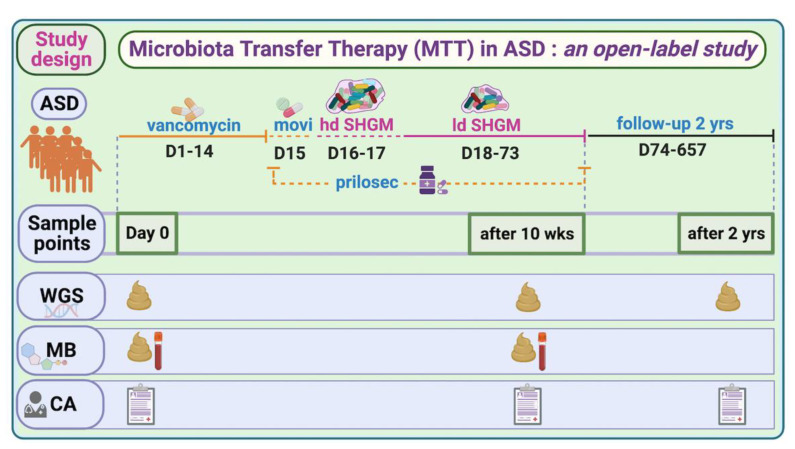
Overview of MTT trial in ASD and study time points for this work. hd—high dose; ld—low dose; SHGM—standardized human gut microbiota; WGS—whole-genome sequencing; MB—metabolomics; CA—clinical assessment (includes all ASD- and GI-associated symptoms; movi—MoviPrep. For complete trial information, refer to [7,11,20].

To investigate the global taxonomical changes before and after MTT (10 wk, 2 yr) in children with ASD (outline of the trial, Figure 1), we used alpha and beta diversity indices (Supplementary Figure S1, Tables S1 and S2). The Shannon index did not show any significant differences when comparing the ASD Baseline (referred to as Baseline) to after MTT or the TD (Supplementary Figure S1A), although the Baseline median for ASD was lower than all other groups (see Supplementary Material 1 for more details). The Jaccard dissimilarity index showed a significant separation (ANOSIM R = 0.45, *p* = 0.001) only between MTT-2 yr and other groups (Supplementary Figure S2A, Table S1A).

Shotgun metagenomic analyses identified a total of 5272 bacterial species in children with ASD (before and after MTT) and TD (Supplementary Tables S1–S5) and univariate comparison between groups showed significant differences in bacterial abundance (see Supplementary Material 1 more details).

### 2.2. MTT Changed Specific Bacterial Species including Fiber-Consuming, Probiotic and Sulfur-Reducing Bacteria

**Figure 2:**
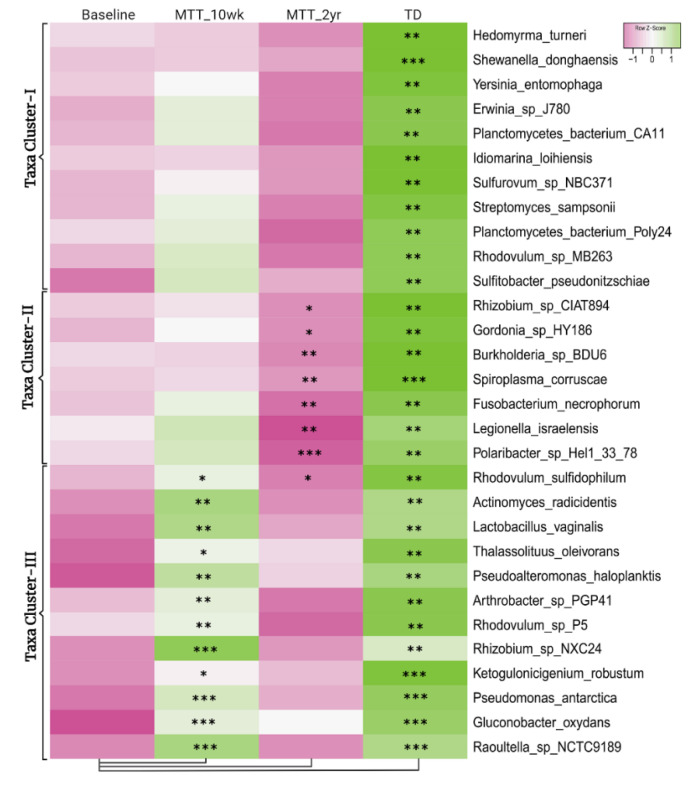
Univariate comparison of the (z-score) relative abundance of gut bacteria for the ASD Baseline group vs. all other groups. Top 30 bacterial species in three clusters that had significantly different relative abundance at Baseline vs. TD (cutoff *p* < 0.01, adjusted *p* < 0.05). Median of bacterial relative abundance was used to construct the heatmap for each group. * Single asterisk indicates *p* < 0.05, ** double asterisks indicate *p* < 0.01, triple *** asterisks indicate *p* < 0.001. All *p*-values are FDR-corrected. ASD: autism spectrum disorder; TD: typically developing.

Figure 2 illustrates the 30 bacteria which had the most significant differences in relative abundance between the ASD and TD groups (cutoff *p* < 0.01, adjusted *p* < 0.05) and showed changes after MTT compared to Baseline in children with ASD. Taxa cluster-I is the group of bacteria that were significantly lower at Baseline compared to TD, and did not change after MTT. Taxa cluster-II were significantly lower at Baseline compared to TD, and decreased further 2 years after MTT. Taxa cluster-III were significantly lower at Baseline, and increased after MTT-10 weeks, but at MTT-2 years they decreased so that there was no difference compared to Baseline except for one that had decreased below Baseline. None of the top 30 bacteria were significantly higher in the ASD group at Baseline compared to TD. Overall, Figure 2 illustrates that the ASD group had significantly lower levels of bacteria, and major increases occurred at MTT-10 weeks and major changes occurred at MTT-2 years. See Supplementary Materials 1 for more details.

**Figure 3:**
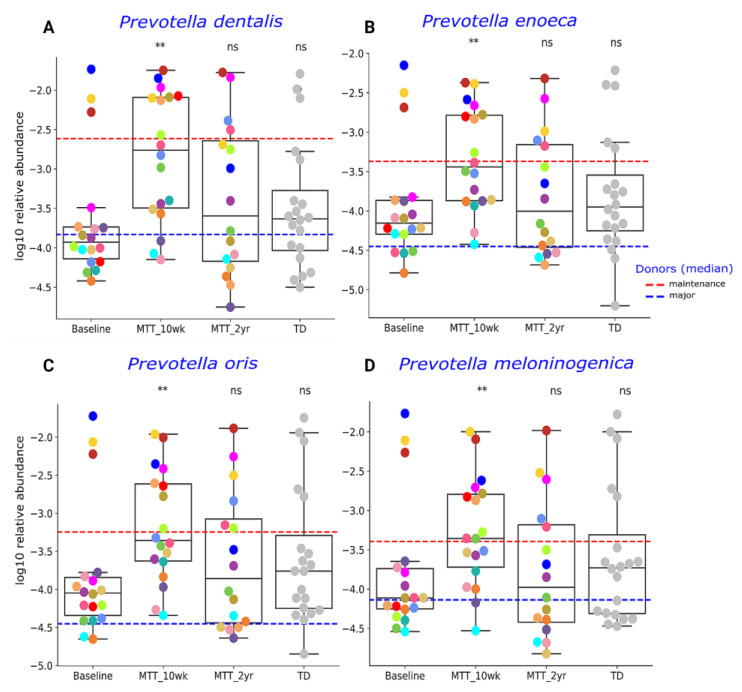
Univariate comparison of the relative abundance (after log_10_ transformation) of (**A**) *P. denatalis*, (**B**) *P. enoeca*, (**C**) *P. oris*, and (**D**) *P. meloninogenica* of ASD Baseline vs. MTT (10 wk, 2 yr) and TD. Red-dashed lines represent the mean of maintenance (*n* = 2) and blue represent median of major donors (*n* = 5). Each colored dot represents one ASD individual and grey-colored dots represent TD. Asterisks represent significant differences between ASD Baseline and the other groups ** double asterisks indicate *p* < 0.01; ns: not significant; all *p*-values are FDR-corrected). ASD: autism spectrum disorder, TD: typically developing.

**Figure 4:**
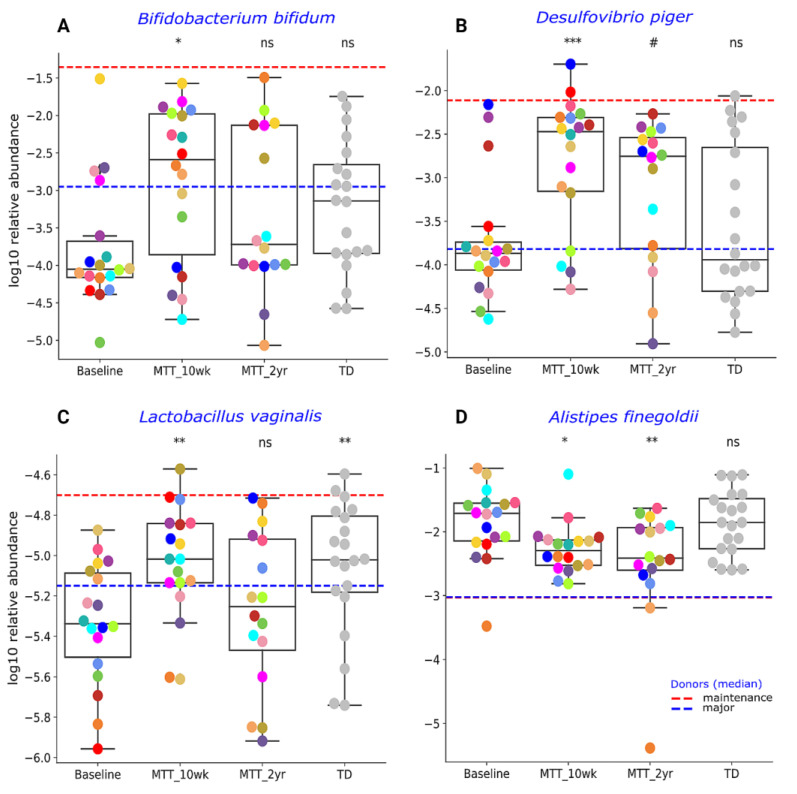
Univariate comparison of the relative abundance (after log_10_ transformation) of different bacterial species: (**A**) *Bifiodobacterium bifidum*, (**B**) *Desulfovibrio piger*, (**C**) *Lactobacillus vaginalis* and (**D**) *Alistipes finegoldii* for ASD Baseline vs. all other groups. Red-dashed lines represent the mean of maintenance donors (*n* = 2) and blue represents the median of major donors (*n* = 5); for *A. finegoldii*, the red and blue lines overlap. Each colored dot represents one ASD individual and grey-colored dots represent TD. Asterisks represent significant differences between ASD Baseline and the other groups (* single asterisk indicates *p* < 0.05; ** double asterisks indicate *p* < 0.01; triple *** asterisks indicate *p* < 0.001; #: statistically significant only after log_10_ transformation; ns: not significant; all *p*-values are FDR-corrected). ASD: autism spectrum disorder; TD: typically developing.

Our initial targeted focus was on *Prevotella* (fiber-consuming bacteria), *Bifidobacterium* (common probiotic), and *Desulfovibrio* (sulfur-reducing), since our prior 16S rRNA gene amplicon study with the same samples found that these genera significantly increased after MTT [7,11]. In this study, we extended our previous findings by examining changes at the species level, and found that ASD at Baseline had lower median levels of specific species of *Prevotella*, *Bifidobacterium* and *Desulfovibrio* compared to TD (raw *p* < 0.05, adjusted *p* > 0.05) (Figure 3 and Figure 4), though it was not statistically significant after FDR correction. As seen in Figure 3 and Figure 4, the relative abundance of several *Prevotella*, *Bifidobacterium* and *Desulfovibrio* species significantly increased (adjusted *p* < 0.05) at MTT-10 wk; at MTT-2 yr, the relative abundance decreased when compared to 10 weeks but remained non-significantly higher than Baseline (median, *p* > 0.05), except for *D. piger*, which remained significantly higher. We identified eight *Prevotella* species that increased significantly after MTT-10 wk, including *P. denatalis*, *P. enoeca*, *P. oris*, *P. meloninogenica* (Figure 3A–D); *P. denticola*, *P. fusca*, *P. intermedia*, and *P. ruminicola* (Supplementary Figure S4A–D). For *Bifidobacterium*, two species increased significantly (*B. bifidum*, (Figure 4A); *B. angulatum*, Supplementary Figure S4E), whereas for *Desulfovibrio*, only *D. piger* increased significantly and remained at higher relative abundance after 2 years (Figure 4B).

Due to higher taxonomical variation (Supplementary Table S3), we focused our analysis on a few specific bacteria (Figure 3 and Figure 4) previously reported as beneficial or associated with ASD or neurological disorders. As seen in Figure 4C, the relative abundance of lactic acid-producing bacteria *Lactobacillus vaginalis* was significantly lower at Baseline vs. TD (adjusted *p* < 0.05), and its relative abundance increased (adjusted *p* < 0.05) at MTT-10 wk compared to ASD Baseline, but decreased somewhat at MTT-2 yr. *Alistipes finegoldii*, which was previously found in other studies to be significantly higher in ASD and linked with ASD [21,22,23], was non-significantly higher (median, *p* > 0.05) at Baseline vs. TD and significantly decreased at MTT-10 wk vs. Baseline (adjusted *p* < 0.05), and remained significantly lower at MTT-2 yr (adjusted *p* < 0.05) in children with ASD and closer to the TD levels (Figure 4D).

### 2.3. Microbial Functional Genes Shifted with MTT

To address the global KEGG Orthologs (KOs) changes before and after MTT (10 wk, 2 yr), we used alpha and beta diversity indices. Shannon index significantly increased after MTT-10 wk compared to ASD Baseline (Supplementary Figure S1B), but after MTT-2 yr, alpha diversity did not change significantly. There was no significant difference in diversity between TD and Baseline. Similar to the taxa analysis, KOs beta-diversity was also significantly distinct for the Jaccard dissimilarity index between MTT-2 yr (ANOSIM R = 0.32, *p* = 0.001) and other groups (Supplementary Figure S2B, Table S1B), suggesting that after 2 years of MTT, rare/less abundant KOs significantly changed in children with ASD compared to Baseline (see Supplementary Material 1 for more details).

**Figure 5:**
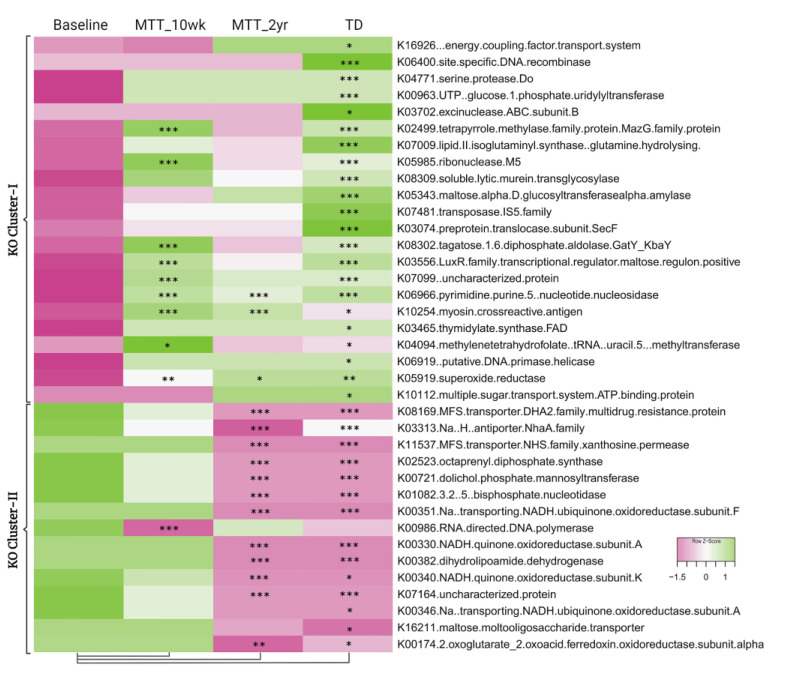
Heatmap of KOs that were significantly lower (KO Cluster-I) or higher (KO Cluster-II) in ASD baseline vs. TD. The heatmap also shows how KOs changed after MTT compared to Baseline, and generally became more similar to the TD group. The median of bacterial relative abundance was used to construct the heatmap for each group. * Single asterisk indicates *p* < 0.05, ** double asterisks indicate *p* < 0.01, triple *** asterisks indicate *p* < 0.001. Univariate statistical comparisons were made for ASD Baseline vs. all other groups. All *p*-values are FDR-corrected. ASD: autism spectrum disorder; TD: typically developing.

To understand and confirm the KOs shift after MTT, we explored specific differences in KOs between sample groups. We identified 5069 KEGG Orthologs (KOs, functional genes) using HUMAnN2. As shown in Figure 5, comparing ASD at Baseline vs. TD, 37 KOs were significantly different (adjusted *p* < 0.05) (Supplementary Tables S3 and S5); 22 KOs were significantly lower (KO Cluster-I), and 15 KOs were significantly higher (KO Cluster-II) (see Supplementary Material 1 more details: Table S6). For KO Cluster-I, nine of the KOs increased at MTT-10 wk and three remained increased at MTT-2 yr. For KO Cluster-II, 1 KO decreased at MTT-10 wk and 12 decreased at MTT-2 yr. Thus, MTT resulted in normalizing many KOs that were initially lower or higher in ASD and became closely similar to TD.

### 2.4. Relative Abundance of Important Metabolic Genes Changed after MTT

**Figure 6:**
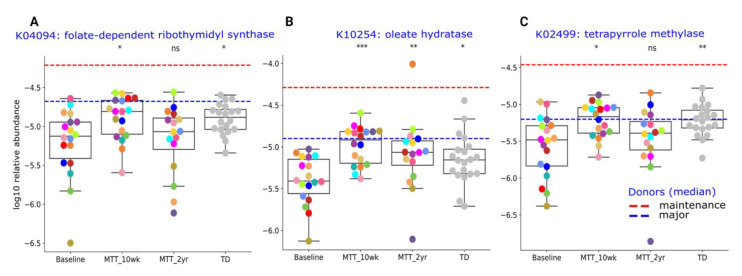
Univariate comparison of the relative abundance (after log_10_ transformation) of gut microbiome genes/KOs that changed significantly after MTT in ASD and became more similar to gene profiles in TD. (**A**) K04094: folate-dependent ribothymidyl synthase, (**B**) K10254: oleate hydratase, (**C**) K02499: tetrapyrrole methylase. Red-dashed lines represent the mean of maintenance (*n* = 2) and blue represent the median of major donors (*n* = 5). Colored dots represent ASD individuals and grey-colored dots represent TD. Asterisks represent significant differences between ASD Baseline and the other groups (* single asterisk indicates *p* < 0.05; ** double asterisks indicate *p* < 0.01; triple *** asterisks indicate *p* < 0.001; ns: not significant; all *p*-values are FDR-corrected). ASD: autism spectrum disorder; TD: typically developing.

Here, we provide more detail on KOs that were significantly different between ASD and TD (adjusted *p* < 0.05), or significantly changed after MTT (adjusted *p* < 0.05), and have been previously potentially linked to ASD. The Supplementary Table S6 lists the metabolic pathways associated with each gene. A closer look at KO Cluster-I (Figure 6) shows changes in relative abundance for genes encoding for: folate biosynthesis (K04094), vitamin B12 synthesis (K02499), and oleic acid synthesis (K10254: omega-9-fatty acids). As seen in Figure 6, the relative abundance of these genes was significantly lower in ASD at Baseline vs. TD and significantly increased (adjusted *p* < 0.05) at MTT-10 wk, and became similar to TD and closer to donor levels. At MTT-2 yr, K04094 and K02499 non-significantly decreased (median, *p* > 0.05), but K10254 remained significantly higher than baseline (see Supplementary Material 1 for more details).

### 2.5. Abundance of Genes Encoding for Oxidative Stress Protection and Sulfur Metabolism Changed after MTT

**Figure 7:**
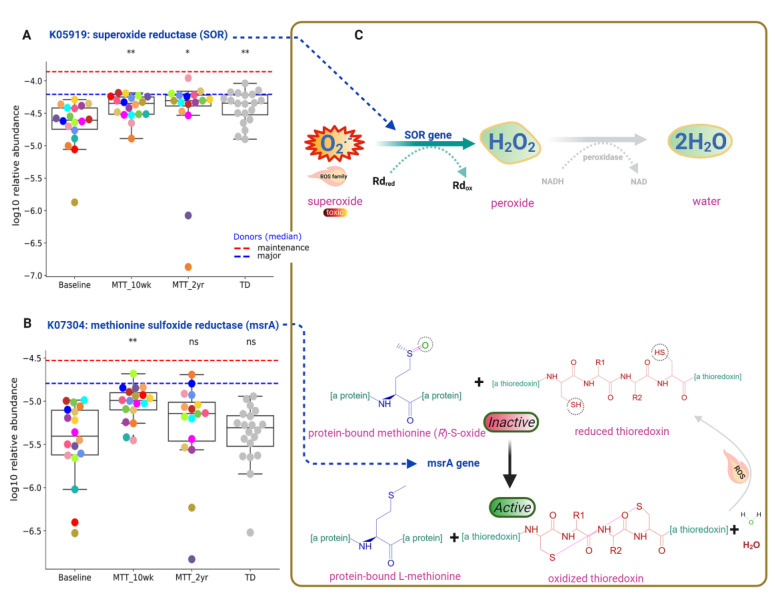
Univariate comparison of the relative abundance (after log_10_ transformation) of gut microbiome genes/KOs that encode enzymes involved in oxygen detoxification and oxidative stress before and after MTT in ASD in comparison with TD. (**A**) Oxidative stress protection and detoxification of reactive oxygen species; K05919 (*dfx* gene, SOR): superoxide reductase. (**B**) K07304 (*msrA*): peptide-methionine (S)-S-oxide reductase. (**C**) Illustration of enzymatic reactions of SOR and *msrA* KOs. Dashed lines represent the median of donors. Red-dashed lines represent the mean of maintenance (*n* = 2) and blue represents the median of major donors (*n* = 5). Colored dots represent ASD individuals and grey-colored ones TD. Asterisks represent significant differences between ASD Baseline and the other groups (* single asterisk indicates *p* < 0.05; ** double asterisks indicate *p* < 0.01; ns: not significant; all *p*-values are FDR-corrected). ASD: autism spectrum disorder; TD: typically developing.

Functional gene analyses of the gut microbiome also revealed that MTT might support the microbial ecosystem by increasing the abundance of microbes that can detoxify oxygen, and microbes with enzymes that protect against oxidative stress. As seen in Figure 5 and Figure 7A, the relative abundance of the gene that encodes for K05919 (*dfx* gene, SOR): superoxide reductase was significantly lower in ASD at Baseline, and significantly increased (adjusted *p* < 0.05) at MTT-10 wk and -2 yr vs. Baseline, and became more similar to the TD group.

This SOR microbial gene encodes for an enzyme that converts toxic superoxide to peroxide, which is subsequently reduced to water, and can help to neutralize ROS similarly to superoxide (Figure 7C, top). Another KO responsible for oxidative stress protection is K07304 (*msrA*): peptide-methionine (S)-S-oxide reductase (Figure 7B). ROS inactivates the sulfur amino acid “methionine-sulfur-oxide”, but KO K07304 (*msrA*) activates methionine-sulfur-oxide by reducing it and oxidizing thioredoxin (Figure 7C, bottom). The relative abundance of KO K07304 (*msrA*) was non-significantly lower at Baseline vs. TD (median, *p* > 0.05), and significantly increased at MTT-10 wk (adjusted *p* < 0.05) (Figure 7B), but there was no significant difference (median remained higher) for MTT-2yr against Baseline. Figure 7C shows the enzymatic reaction of K05919 (SOR) and K07304 (*msrA* gene) enzymes for oxygen detoxification and oxidative stress protection after MTT, respectively.

**Figure 8:**
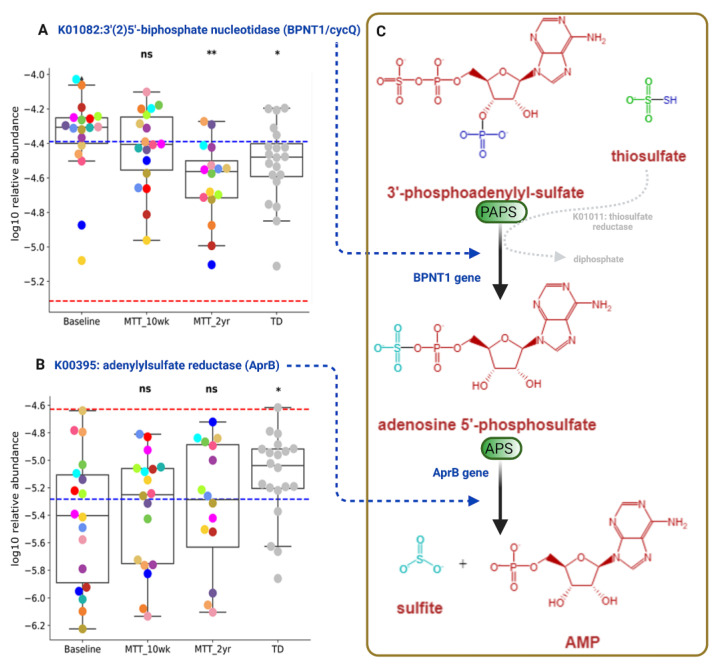
Univariate comparison of the relative abundance (after log_10_ transformation) of genes encoding for enzymes involving microbial sulfur metabolism (dissimilatory sulfate reduction) before and after MTT in ASD in comparison with TD. (**A**) K01082 (*BPNT1/cycQ*) 3′(2), 5′-bisphosphate nucleotidase, (**B**) K00395 (*aprB*) adenylylsulfate reductase, subunit B. (**C**) Diagram Illustrating dissimilatory sulfur reduction and the contribution of *BPNT1* and *aprB* to the process. Each colored dot represents an ASD individual and grey-colored dots represent TD. Red-dashed lines represent the mean of maintenance (*n* = 2) and blue represents the median of major donors (*n* = 5). Asterisks represent significant differences between ASD Baseline and the other groups (* single asterisk indicates *p* < 0.05; ** double asterisks indicate *p* < 0.01; ns: not significant; all *p*-values are FDR-corrected). ASD: autism spectrum disorder; TD: typically developing.

3′-Phosphoadenosine-5′-phosphosulfate (*PAPS*) and adenosine-5′-phosphosulfate (APS) are important phosphosulfate compounds for sulfur metabolism that participate in assimilatory and dissimilatory sulfate reduction via sulfate-reducing bacteria (Supplementary Figure S5) [24,25]. As shown in Figure 8, the relative abundance of the gene that encodes for KO K01082 (*BPNT1/cycQ*) 3′(2′), 5′-bisphosphate nucleotidase, the enzyme that converts PAPS to APS, was significantly higher at Baseline and non-significantly decreased (median, *p* > 0.05) at MTT-10 wk, and decreased significantly at MTT-2 yr vs. Baseline (adjusted *p* < 0.05) and its relative abundance became more similar to TD (Figure 8A).

The relative abundance of the gene that encodes for KO K00395 (*aprB*) adenylylsulfate/APS reductase, subunit B (the enzyme that converts APS to sulfite), was significantly lower in ASD Baseline vs. TD (adjusted *p* < 0.05), and non-significantly increased after MTT-10 wk and -2 yr (median, *p* > 0.05), and became more similar to the TD group (Figure 8B).

Figure 8C illustrates dissimilatory sulfur reduction and the contribution of *BPNT1* and *aprB* to the process.

We also explored differences in the relative abundances of important functional genes that were not significantly different between Baseline and TD but changed significantly after MTT-10 wk and -2 yr (adjusted *p* < 0.05) (Supplementary Figure S6).

### 2.6. Correlation Analysis Shows Links between Omics and GSRS

Correlation analysis was used to uncover the potential relationship of bacterial taxonomy, bacterial genes, plasma metabolites [20], and ASD and GI symptom data (Supplementary Figures S7 and S8). Within the correlation network, we identified 280 nodes with 420 edges with correlations of R > 0.6 or R < −0.6, and adjusted *p* < 0.05. Network analysis was performed only for the ASD Baseline group and only included blood metabolites, not fecal metabolites, as we did not observe significant changes in fecal metabolites after treatment [20].

The correlation network showed 241 positive (green color) and 179 negative (red) interactions/correlations for genes (KOs) related to neurotransmitters/neuroactive molecules, amino acids, indole, taurine, tyramine derivatives, and for sulfur metabolism (Supplementary Material 1, Figures S7 and S8). We did not observe significant correlation networks for *Prevotella*, *Bifiobacterium*, or *Desulfovibrio* species, or important KOs for oxidative stress, dissimilatory sulfate reduction or other taxa/KOs mentioned in this section.

## 3. Discussion

The primary hypothesis for symptom (ASD, GI) improvement with MTT was the remodeling of the gut microbiome to resemble more gut microbiomes of healthy donors or TD/controls [26,27]. Taxonomic analysis showed many lower abundances of taxa at Baseline that increased after MTT-10 wk (Figure 2). Interestingly, after 2 years, the taxonomic composition became distinct to the other groups analyzed (Figure 2, Supplementary Figure S2A), suggesting MTT changed the microbial composition, but over time these children developed different/own microbial composition.

Previous 16S rRNA gene amplicon measurements of the same samples used in this study showed a lower abundance of the genera *Prevotella*, *Bifidobacterium* and *Desulfovibrio* before treatment, and major improvements after MTT [7,11]. Therefore, we focused first on these bacteria. At Baseline, *Prevotella* sp. abundance compared to TD (Figure 3) was lower, although not statistically significantly (*p* > 0.05), but the relative abundance increased significantly at MTT-10 wk vs. Baseline in eight species (adjusted *p* < 0.05) was consistent with our previous findings at genera level using 16S rRNA gene amplicon sequencing [11]. Previous research studies have reported lower or depleted levels of *Prevotella* in ASD compared to TD in fecal [17,23,28,29] and oral microbiota [30]. We identified many *Prevotella* species in fecal samples, but some of them were similar to oral *Prevotella species*, such as *P. dentalis* (Figure 3) and *P. denticola* (Supplementary Figure S4A). We hypothesize that these oral *Prevotella* may have transferred from mouth to gut more than normal because of the use of a proton pump inhibitor in our MTT study [7]. Many gut *Prevotella* species are fiber-consuming bacteria [31,32]. One possible reason for *Prevotella*’s depletion in children with ASD in the USA could be lower fiber content in the Westernized diet leading to the depletion of bacteria with fiber-degrading enzymes [9,33], and this could contribute to poorer GI health and behaviors in children with ASD [17].

Similarly, two probiotic examples of *Bifidobacterium (B. bifidum* Figure 4A; *B. angulatum*, Supplementary Figure S4E) and a sulfur-reducer (*Desulfovibrio piger*, Figure 4B) significantly increased at MTT-10 wk vs. Baseline and their relative abundance became comparable to TD (Figure 4A,B). This is also consistent at the genera level with our 16S rRNA gene amplicon study [7,11], but the abundance of these microbes non-significantly increased (median) at MTT-2 yr compared to Baseline (*p* > 0.05) (Figure 4A,B). The depletion of *Bifidobacterium* species has been reported widely in ASD [8,23,29,34,35]. *Bifidobacteria* are SCFA producers, and “psychobiotics” [36], and they modulate the gut–brain signals via *γ*-aminobutyric acid (GABA) and glutamate metabolism [37]. *Bifidobacterium* have been reported to improve behavior and prevent depression-like behaviors in mice [38,39,40]. *Desulfovibrio* is a sulfur-reducing bacteria (SRB) that can degrade mucins, SCFAs, and amino acids (glutamate, alanine) in the human colon, and may help to maintain the integrity of the gut epithelium [41,42]. However, some reports have shown a seemingly different result: higher *Desulfovibrio* in ASD Baseline compared to TD fecal samples [43,44,45]. It is important to note that *Prevotella, Bifidobacterium and Desulfovibrio* species were not significantly different between ASD Baseline and TD, although the median was lower for Baseline than TD. After MTT (10 wk), the abundance of these bacteria significantly increased and moved closer to the maintenance donor (dashed-red horizonal line) (Figure 3 and Figure 4).

The relative abundance of *Lactobacillus vaginalis* increased significantly at MTT-10 wk vs. Baseline and became more similar to TD (Figure 4C). *L. vaginalis* is a common vaginal commensal [46], and has also been reported in human feces and oral cavities [47]. Similar to *Bifidobacterium*, the genus *Lactobacillus* are also “psychobiotics” and can modulate the gut–brain connection via neuroactive molecules (GABA, glutamate) [36,37].

A higher relative abundance of *Alistipes* species has been reported in children with autism, pervasive developmental disorder not otherwise specified (PDD-NOS) [23], and depression [22]. Researchers have postulated that *Alistipes* may disrupt the gut–brain axis by decreasing serotonin (indole-positive organism) [21,22] and impair cognition by producing propionic acid in rats [48]. Interestingly, the relative abundance of *Alistipes finegoldii* was significantly lower after MTT (10 wk and 2 yr) compared to Baseline (Figure 4D) and became comparable to TD. It is important to note that, after MTT (10 wks), bacterial abundance changed, but over time (2 yrs) returned closer to Baseline levels for all presented taxa except *Alistipes* and *Desulfovibrio* (Figure 3 and Figure 4). This finding suggests that the impact of MTT treatment in these bacteria were temporary and longer treatment may have been needed. *Alistipes* and *Desulfovibrio* remained significantly changed after 2 years of MTT (Figure 4).

At the global level of KOs, we observed many altered levels of microbial genes at Baseline compared to TD, but after MTT, microbial gene abundance became similar to TD or closer to healthy donors (Figure 5). This finding suggests that MTT had a positive impact on ASD children and helped to restore the metabolic pathways. To understand this more closely, we looked at specific KOs. Alterations in folate metabolism and folate/vitamin B9 deficiency have been linked with ASD [49]. Interestingly, *trmFO* (K04094), the gene that encodes for the tetrahydrofolate-synthesizing enzyme, was at lower abundance in Baseline vs. TD, but after MTT, its relative abundance significantly increased and became more similar to the TD group (Figure 6A). This suggests that microbes enhanced by MTT have the potential to synthesize folate for the host, and folate supplementation has been demonstrated to improve ASD symptoms. It has been hypothesized that lower levels of *Prevotella* and *Bifidobacterium* (Figure 3A–D and Figure 4A) may lead to reduced levels of folate production due to diminished folate-dependent remethylation in ASD [24].

Oxidative stress is a major concern in ASD etiology [50] and it can disrupt neuron connections in the brains of individuals with ASD by causing neuroinflammation [50,51] and cognitive impairment [50,51]. Imbalance in redox reactions and a lack of antioxidants such as folinic acid, glutathione, vitamins (C and E) or coenzymes NAD^+^/NADH are possible contributing factors for ROS in ASD [50,52,53]. Interestingly, the relative abundance of genes that encode for K05919: superoxide reductase (*dfx* gene, SOR) was low at Baseline vs. TD, but significantly increased after MTT (10 wk, 2 yr), and its abundance became similar to TD (Fig 7A). This microbial SOR converts highly reactive and toxic superoxide (O_2_^-^) to less toxic hydrogen peroxide (H_2_O_2_), which is subsequently converted to H_2_O [54,55]. SOR is present in anaerobic microbes such as SRB, e.g., *Desulfovibrio* [54,56,57]. Interestingly, we found that the relative abundance of *Desulfovibrio piger* was low at Baseline but significantly increased after MTT (Figure 4B).

Researchers have reported sulfate deficiency or altered sulfate metabolism as being linked with autism [8,24,58,59]. We observed differences in relative abundance in genes that encode for two KOs that participate in microbial dissimilatory sulfate reduction: K01082 (*BPNT1/cycQ*) 3′(2′), 5′-bisphosphate nucleotidase and K00395 adenylylsulfate reductase, subunit B (*aprB gene*) (Figure 8, Supplementary Figure S5). We also observed a significant negative correlation of K01082 (*BPNT1/cycQ*) with sulfur-reducing bacteria *Selenomonas species* (Supplementary Figures S9A and S10B (relative abundance)) and with *Desulfovibrio piger* (Supplementary Figure S9B), although the effect size was small. These findings suggest an imbalance in microbial sulfate reduction in children with ASD that is normalized by MTT, bringing these KOs levels close to the levels observed in TD [25]. Our above findings suggest that ASD at Baseline had an altered abundance of microbial genes for dissimilatory sulfate reduction (sulfur metabolism, Supplementary Figure S5). We hypothesize that MTT had a positive impact on microbial sulfur metabolism, and the relative abundance of genes involved in sulfur metabolism in ASD children became more similar to TD in the after-MTT group. Another interesting finding was that after MTT, the relative abundance of the functional genes in ASD children became more similar to the relative abundance of the donors.

Correlations of bacteria with GI and ASD symptoms were investigated. *Nostoc linckia*, a cyanobacteria, was positively correlated with GSRS at Baseline (Supplemental Figures S7 and S10A). It has been suggested that some *Nostoc* species can produce β-*N*-methylamino-L-alanine (BMAA) and target the gut immune system and cause chronic low-grade inflammation [60]. However, no significant correlations between CARS and microbiota were observed in this study. For the correlation in this study, only plasma metabolite data were used from Kang et al. [20], not including fecal metabolites. Fecal metabolites were largely unchanged after MTT, as described in our previous study [20]. We did not find a statistically significant correlation of microbiomes, neither with demographic measurements such as age nor with stool consistency. Dietary information was not included in the correlation analysis, as limited diet information was available after MTT in children with ASD.

A strength of this study is that it is the first study exploring the gut microbiome and metabolic pathways in children with ASD before and after MTT using shotgun metagenomic data analyses. This study provides higher taxonomic classification and an indication of microbial pathways that might be important to achieve improvements through microbial interventions such as MTT. k-mer (short sequencing fragments) alignment was used to process sequences and the marker gene database was used for taxonomic and functional analysis. Three of the eighteen children did make changes in their diet in the two years after MTT treatment ended [11]. The TD cohort did not have 10-week and 2-year follow-up time points, and the study had a small sample size and open-label design, as described in Kang et al. [7,11]. Since ASD and GI symptoms improved after MTT, we hypothesize that MTT led the primary improvement in symptoms in children with ASD, and diet might have a complementary impact after children are more comfortable.

## 4. Materials and Methods

### 4.1. Outline of the Trial

This shotgun metagenomic study is an extension of a phase-1 open-label study of microbiota transfer therapy in children with ASD, previously published for 16S rRNA gene amplicon sequencing [7,11]. In brief, we recruited 20 TD children and 18 children with ASD within the age range of 7–16 years. During the 10 weeks of the trial, the ASD group was treated with vancomycin for 2 weeks, given a one-day bowel cleanse with MoviPrep, then 1–2 days of high-dose liquid microbiota (Major donor) and 7–8 weeks of low-dose liquid microbiota (maintenance donor) and Prilosec (stomach acid suppressant) (Figure 1). A follow-up evaluation was conducted at 8 weeks post-treatment (i.e., 10 weeks from Day 0), and at 2 years post-MTT (only 16 out of 18 provided fecal samples) [11]. All participants’ characteristics and their medical and diet history were recorded, as described in Kang et al. [7,11]. There was no change in diet during the MTT treatment, and after two years of treatment diet was recorded, as described in Kang et al. [11].

### 4.2. Metagenomics Sequencing

Fecal DNA extracted from a previous study was used for sequencing [11]. The DNA samples were from three distinct timepoints: ASD Baseline (*n* = 18), at the end of the 10-week treatment (MTT-10 wk, *n* = 18), and at the 2-year follow-up (MTT-2 yr, *n* = 16). Samples were also collected from the TD (*n* = 20), major donor (*n* = 5) and maintenance donor (*n* = 2) cohorts. For shotgun metagenomics, DNA was sequenced on the Illumina NextSeq 500 platform (Illumina, CA, USA) to generate 2 × 150 bp paired-end reads at greater sequencing depth with a minimum of 10 million reads.

### 4.3. Sequencing Analysis

We received 28,470,588 ± 9,835,000 (mean ± SEM) reads per sample from shotgun metagenomic sequencing. The quality of raw reads was examined with MultiQC [61]. Adapters from the reads and low-quality reads with length < 50 bp or phred < 30 were removed. To avoid human genome contamination, we mapped all the reads against the UCSC Genome Browser’s hg38 human genome reference database using a Burrows–Wheeler aligner (bwa) [62] and discarded mapped reads. Reads that were declared unmapped (without human genome) were used for downstream analyses.

Bacterial taxonomic composition was characterized using Kraken2 (v2.0.7, https://github.com/DerrickWood/kraken2) with the NCBI RefSeq database [63]. Kraken2 is an ultrafast taxon-assigning tool that uses the exact alignment of *k*-mers in a sequence and then finds the LCA (lowest common ancestor) taxa by comparing against the database. Following taxa assignment, the species-level sequence abundance estimation algorithm Bracken (Bayesian re-estimation of *Abundance* with KrakEN, v2.6) [64] was used to re-estimate the abundance of assigned taxa and calculate their relative abundance. Our taxonomic assignment was for bacterial phylotypes. This work uses “bacteria” or “bacterial species” terms for “phylotypes” throughout the text.

HUMAnN2 (the HMP unified metabolic analysis network, v2, https://github.com/biobakery/humann/tree/2.9) was used to identify the functional genes/pathways associated with microbiome gene markers [65]. HUMAnN2 works with MetaPhlAn2 (Metagenomic Phylogenetic Analysis, v2, https://github.com/biobakery/MetaPhlAn2) and its ChocoPhlAn pangenome database, and uses the MetCyc, MinPath and UniRef90 databases [65]. From HUMAnN2 output, gene family abundance that had a 90% match with UniRef90 were used to convert this information to KOs (KEGG Orthologs: functional genes). Subsequently, the relative abundance was calculated using all KOs’ absolute abundance for each sample.

### 4.4. Fecal and Plasma Metabolomics

An untargeted metabolomics approach was previously performed by Metabolon Inc. to measure fecal and plasma metabolites using ultra-high-performance liquid chromatography tandem mass spectroscopy (UHPLC-MS/MS) (https://www.metabolon.com) (accessed on 10 January 2022). Sample preparation, metabolite measurement and results are described in our prior work [20]. In this study, we leverage metabolite data from Kang et al. [20] to perform downstream analyses with shotgun metagenomics. See Supplementary Material 1 for more information.

### 4.5. GI and ASD Symptom Assessment

All GI and ASD symptom measurements are described in detail in [11]. In brief, for GI symptoms, a revised version of the Gastrointestinal Symptom Rating Scale (GSRS) with the five domains of Abdominal Pain, Reflux, Indigestion, Diarrhea, and Constipation was used. Daily stool records (DSR) using the Bristol Stool Form scale were also recorded. For ASD symptoms, Parent Global Impressions–III (PGI-III), Childhood Autism Rating Scale (CARS), Aberrant Behavior Checklist (ABC), Social Responsiveness Scale (SRS), and Vineland Adaptive Behavior Scale II (VABS-II) measurements were taken for all ASD participants.

### 4.6. Diversity Index Calculation

To explore the taxonomy and KO’s changes before and after MTT, alpha-diversity (Shannon index) and beta-diversity (Jaccard and Bray–Curtis dissimilarity indices) were calculated. Initially, taxonomical and KO abundance data were imported to Qiime2 (v2022.2) [66] for calculation of the diversity indices. For the Shannon diversity index, univariate analysis was performed (Wilcoxon signed rank test for paired and Mann–Whitney tests for unpaired) and visualized using the *ggpubr* R package (v0.4.0). For beta-diversity, pairwise ANOSIM (analysis of similarities) with 999 permutations was used for the statistical comparisons in Qiime2 and visualized using the Dokdo API (https://github.com/sbslee/dokdo, v1.14.0) in python with Qiime2. *P*-values were corrected with the Benjamini–Hochberg method [67] and assigned as q-values.

### 4.7. Multi-Omics Correlation Network

To understand the interaction between bacteria, metabolites, and functional gene abundance with GI and ASD symptoms, a correlation network was derived. To make the network, relative abundances of bacteria and KOs, metabolites in Z-scores, and GI and ASD symptom severity scale data were used for the correlations. No additional data transformation was employed for the correlations. The relationships between variables were characterized as being meaningful using the Pearson’s correlation coefficient (R > ±0.6, *p* < 0.05) subjected to leave-one-out FDR correction (*p* < 0.05). FDR-corrected correlation coefficients and *p*-values for each variable were subjected to Cystoscope (v3.8.2) to construct and visualize the correlation network. Unconnected nodes were excluded from the network. In this study, only plasma metabolites data were used from Kang et al. [20], as fecal metabolites did not show significant change before and after MTT.

### 4.8. Statistical Analysis and Plots

Univariate analysis comparing the sample distributions was performed for taxa and pathway data via hypothesis testing with false discovery rates determined using the leave-one-out approach. The issue of multiple hypothesis testing was addressed by determining the false discovery rate (FDR) for each significant finding (*p* < 0.05) using a leave-one-out approach and considering *p* < 0.05 as significant. In each group, more than 20% of samples with zeros in relative abundance (for both taxa and KOs) were filtered out during univariate analysis (details in Supplementary Material 1). Since this is a pilot study, we are reporting some non-significant (*p* > 0.05) changes that are of possible interest and using the term “non-significantly” throughout the manuscript.

All taxonomical and KOs plots were made with Python (v3.8.5) in Jupyter Notebook (v6.1.4) using numpy (v1.19.2), matplotlib (v3.3.2), seaborn (v0.11.0), pandas (v1.1.3), scipy (v1.5.2), etc. The area under the receiver operating characteristic (AUROC) was calculated (Supplementary Material 1) in MATLAB (v2018B). For metabolic pathway images, MetaCyc (Metacyc.org) was used [68]. BioRender (Licensed, https://biorender.com) (accessed on 15 September 2022) and Inkscape (v1.1) were used to create or edit the figures.

## 5. Conclusions

**Figure 9:**
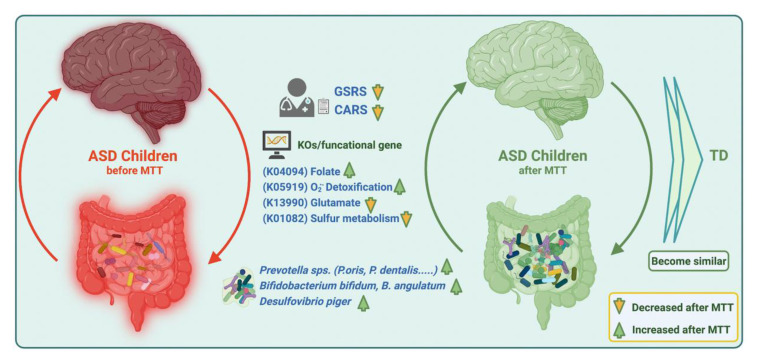
Overview illustration of microbiome and metabolic pathways/KOs in children with ASD before and after MTT. Gastrointestinal Symptom Rating Scale (GSRS); Childhood Autism Rating Scale (CARS).

Our current findings suggest that MTT in ASD children changed the microbial composition by normalizing levels of many bacteria that were initially low. MTT also increased the abundance of previously detected beneficial bacterial such as *Prevotella*, *Bifidobacterium*, and sulfur-reducer *Desulfovibrio* at the species level, but over the time (2 yrs) the abundance of *Prevotella* and *Bifidobacterium* decreased, which suggests a longer MTT treatment time or a booster after a certain time might be needed for the retention of these bacteria. Similarly, MTT also resulted in normalizing the levels of many bacterial genes (KOs). Interestingly, microbial metabolic genes (KOs) for folate biosynthesis, oxidative stress protection and sulfur metabolism were different at ASD Baseline than TD, but after MTT (10 wk, 2 yr), they became more similar to the TD and/or donor levels (Figure 9). We recommend further mechanistic in vitro experiments and metatranscriptomics studies with larger cohorts to understand the role of these specific microbes, functional gene expression, and metabolites in ASD before and after MTT.

## Data Availability

The data presented in this study are openly available in the NCBI SRA repository under BioProject ID PRJNA782533 and can be accessed at https://www.ncbi.nlm.nih.gov/bioproject/?term=PRJNA782533.

## Supplementary Materials

The following supporting information can be downloaded at: https://www.mdpi.com/article/10.3390/ijms232113481/s1, Figure S1: The alpha-diversity before and after MTT in children with ASD; Figure S2: Jaccard distance dissimilarity index before and after MTT in children with ASD; Figure S3: The beta-diversity before and after MTT in children with ASD; Figure S4: Univariate comparison of the relative abundance (after log10 transformation) of different taxa; Figure S5: Overview of sulfur metabolism; Figure S6: Univariate comparison of the relative abundance (after log10 transformation) of gut microbiome genes/KOs that changed significantly after MTT in ASD but did not become similar to TD; Figure S7: Network analyses of microbes, microbial metabolic pathways, plasma metabolites and ASD symptoms for children with ASD at Baseline; Figure S8: Subset of correlation network between microbiome and pathways with plasma metabolites; Figure S9: Correlation tests between taxa and microbial genes; Figure S10: Univariate comparison of the relative abundance (after log10 transformation) of different taxa; Table S1: Beta Diversity: Jaccard dissimilarity index; Table S2: Beta Diversity: Bray-Curtis dissimilarity index; Table S3: Taxonomy and KOs comparison between groups; Table S4: Differences in number of taxa between groups; Table S5: Taxonomy and KOs comparison between TD and all ASD groups; Table S6: List of significantly different 37 KOs/metabolic pathways in ASD Baseline vs. TD children from Figure 5.
