# Supplementary material for: Shotgun Metagenomics Study Suggests Alteration in Sulfur Metabolism and Oxidative Stress in Children with Autism and Improvement after Microbiota Transfer Therapy
Source: Int J Mol Sci. 2022 Nov 3;23(21):13481. doi: 10.3390/ijms232113481 (PMC9654974; doi:10.3390/ijms232113481)
Supplement: Supplementary file 1 [file ijms-23-13481-s001.zip › ijms-1948060-supplementary.pdf]

Shotgun metagenomics study suggests alteration in sulfur metabolism and oxidative stress in children with autism and improvement after microbiota transfer therapy

Khemlal Nirmalkar[1*], Fatir Qureshi[2,4], Dae-Wook Kang[1,#], Juergen Hahn[2,3,4], James B. Adams[5], Rosa Krajmalnik-Brown[1,6,,*]

[1] Biodesign Center for Health Through Microbiomes, Arizona State University, Tempe, AZ, USA

[2]Department of Biomedical Engineering, Rensselaer Polytechnic Institute, Troy, NY 12180, USA

[3]Department of Chemical and Biological Engineering, Rensselaer Polytechnic Institute, Troy, NY 12180, USA

[4]Center for Biotechnology and Interdisciplinary Studies, Rensselaer Polytechnic Institute, Troy, NY 12180, USA

[5]School for Engineering of Matter, Transport, and Energy, Arizona State University, Tempe, AZ 85287, USA

[6]School of Sustainable Engineering and the Built Environment, Arizona State University, Tempe, AZ 85281, USA

[#]Current address: Department of Civil and Environmental Engineering, The University of Toledo, Toledo, OH 43606, USA

[*]Correspondence: Rosa Krajmalnik-Brown Dr.Rosy@asu.edu and Khemlal Nirmalkar_khem@asu.edu

Supplementary Material:

Material and Methods

Fecal and Plasma Metabolomics: An untargeted metabolomics approach was performed by Metabolon Inc. to measure fecal and plasma metabolites using ultrahigh performance liquid chromatography-tandem mass spectroscopy (UHPLC-MS/MS) (https://www.metabolon.com). Sample preparation and metabolites measurement are described in our prior work,[1]. In brief, peak area integration and relative intensity was used to measure the metabolites and were normalized such that the median was set equal 1. Imputation was performed for missing values by taking the lowest value of each measured metabolite divided by the square root of 2. As a threshold for missing values, we considered the presence of metabolites minimum in 39% of our samples. Lower than this threshold, metabolites were excluded for the analysis. Metabolomic analysis was done for ASD at baseline, at the end of MTT-10wks, and for the TD group. While collected, measurements taken at the 2-years follow up timepoint could not be compared to prior samples due different samples batch preparation between before and after 2 years of MTT.

Statistical analysis & plots: Univariate analysis comparing the sample distributions was performed for taxa and pathway data *via* hypothesis testing with positive false discovery rates (FDR) determined using a Bayesian leave-one-out approach. This method was chosen due to the sample size relative to the number of hypotheses tested. Compared to the Benjamini-Hochberg (BH) method, the FDR technique has been demonstrated on average to achieve a higher statistical power. Furthermore, the assumptions of the BH approach were not satisfied due to the correlated nature of the microbiota abundance measurements. The issue of multiple hypothesis testing was addressed by determining the FDR for each significant finding ($p< 0.05$) using a leave-one-out approach and considering $p<0.05$ as significant. More than 20% of samples with zeros were filtered out from univariate analysis for taxa and KOs.

When comparing unpaired data, such as between the TD and ASD baseline cohorts, both sample sets were initially tested for normality. D'Agostino and Pearson's test were used for evaluating both sample sets separately. Dependent on if the normality assumption was accepted or rejected, the samples were either compared using an F-test or 2-sample Kolmogorov-Smirnov test, respectively. The F-test was used when both sample sets were drawn from normal distributions, and this test was performed to determine if the variance was significantly different between the two groups. In the case in which the normality assumption likely held true (p-value>0.05) and there was equal variance, the 2-sample t-test was performed. In the situation when normality was observed and the variance was observed to be unequal, a Welch's test was used.

Alternatively, if one or both sample sets were evaluated and determined to be derived from a non-parametric distribution, the 2-sample Kolmogorov-Smirnov test was used to determine if the same underlying non-parametric distribution was present for both. A Mann-Whitney test was used if the two groups were observed to follow the same non-parametric distribution. In cases where different distributions were observed between samples, both sample sets were adjusted by their means and then the 2-sample Kolmogorov-Smirnov test was used again to evaluate the distribution structure. The Welch's test was used if both sample sets were still tested to be derived from different distributions, and the Mann-Whiney test was used if the distributions were determined by testing to be similar.

Taxa and pathway/KOs measurements taken post-treatment at the MTT-10wk and MTT-2yr time points were analyzed and compared to their baseline counterparts *via* optimized paired hypothesis testing as well. Normality testing *via* the D'Agostino and Pearson's test was used to determine whether to use a parametric or non-parametric test. When the normality assumption held for both sample sets, a paired t-test was performed. Alternatively, when either sample set was not normally distributed, the

Wilcoxon matched-pairs signed rank test was used. All univariate comparisons were made for all groups against baseline.

To account for multiple hypothesis testing, the false discovery rate was determined using a leave-one-out method. This technique proceeds iteratively on each data entry, i.e., the measurements taken for the pathway or taxa data. Using the same test selection protocol outlined above for both the paired and unpaired variants, the p-value is recalculated with one individual's measurements excluded from the analysis. This is repeated such that every combination with one sample being excluded is assessed. The FDR for each variable is the ratio of the number of p-values greater than 0.05 to the total amount of p-values that were evaluated. A finding was deemed statistically significant if the FDR value determined was under 0.10.

Results:

To address the global taxonomical and KOs changes before and after MTT (10wk, 2yr), we used alpha and beta diversity indices. This included the Shannon index (Figure S1) and Jaccard index (Figure S2). For the bacterial diversity, unlike Jaccard, no distinct separation was observed between all groups for Bray-Curtis distance (weighted measurement) (Figure S3A, Table S2A), though overall distance between all groups was significant (ANOSIM R=0.14, p=0.001), suggesting dominant taxa were not significantly different between all groups. For KOs beta-diversity, no distinct separation was observed between all groups for Bray-Curtis distance (Table Figure S3B, Table S2B), though overall distance between all group was significant (ANOSIM R=0.16, p=0.001). Bray-Curtis results suggest that dominant taxa and KOs did not change significantly in ASD children after MTT and also not significantly different compared to TD.

Shotgun metagenomic analyses revealed 5272 unique bacterial species in children of the ASD and TD cohorts (Table S1-S3). Comparing the sequences in the ASD cohort at Baseline *vs*. TD, we Identified 371 bacterial species that had significantly lower relative abundance (adjusted p<0.05) compared to the TD group, and none were significantly higher. For these 371 bacterial taxa that were initially lower in the ASD cohort, after MTT-10wks, the relative abundance of 98 (out of 371) bacterial species significantly changed (adjusted p<0.05); 97 significantly increased and 1 decreased (*Alistipes finegoldii*) in the MTT-10wks group compared to Baseline. However, at MTT-2yrs the relative abundance of 60 (out of 371) bacterial species were significantly decreased (adjusted p<0.05) compared to Baseline, becoming less similar to the TD cohort (Table S1-S3).

When analyzing all 5272 species, the longitudinal pairwise comparison between Baseline and MTT-10wks showed 666 taxa were significantly different; abundance of 650 bacterial species increased and 16 decreased significantly (adjusted p<0.05, Table S1). After MTT-2yrs, 1611 taxa were significantly different to baseline (adjusted <0.05);12 increased (Table S1-S2) and 1599 decreased in MTT-2yrs compared to Baseline. This suggests that MTT initially led to an increase in relative abundance of 650 species, but at 2 years only 12 had higher relative abundance. Similarly, MTT initially decreased the relative abundance of only 16 species, but at 2 years 1599 were at lower relative abundance. Comparison of TD *vs*. MTT data (10wks, 2yrs) for all bacteria showed similar trend to ASD Baseline *vs*. MTT (Table S1-S2). So, initial changes occurred after MTT-10wks, but additional major changes had occurred at 2-years post-treatment. Although changes led initially to more similarity to the TD group, at the 2-year follow-up the ASD group had formed a microbiome that was very distinct from its initial Baseline, and was distinct from the TD group (Table S1-S2).

Comparing data of TD *vs*. MTT-10wks, 435 bacterial species were significantly different (adjusted p<0.05); 48 were higher and 387 were lower in MTT-10wks. For TD *vs*. MTT-2yrs, 2990 bacterial species were significantly different (adjusted p<0.05); 6 were higher (Table S2-S3) and 2984 were lower after MTT-2yrs compared to TD (Table S2-S3). The above findings suggest that bacterial abundance became less similar after MTT-2yrs compared to TD, so after 2-years ASD children developed their own different gut microbiome compared to all other groups.

Figure 2 illustrates the differences in the top 30 differentially abundant between ASD and TD groups (cutoff p<0.01, adjusted p<0.05) and changes after MTT compared to Baseline in children with ASD. Taxa Cluster-I shows the 11 bacteria which were significantly lower (cutoff p<0.01, adjusted p<0.05) in the Baseline group compared with the TD group. At MTT-10wk, bacterial abundance slightly increased but no statistically significant change was observed compared to Baseline. However, at 2 years the relative abundance had generally decreased (non-significantly, p>0.05) back to relative abundance at Baseline. Note that there were no bacteria that were significantly higher at Baseline in the ASD group compared to the TD group.

Taxa Cluster-II shows the 7 bacterial taxa with significantly lower (cutoff raw p<0.01, adjusted p<0.05) in relative abundances at Baseline group compared to TD, and which did not change significantly at MTT 10 weeks, but significantly decreased (adjusted p<0.05) at MTT-2yr compared to Baseline.. Taxa Cluster-III shows the 12 bacteria whose relative abundances were significantly lower (cutoff raw p<0.01, adjusted p<0.05) in the Baseline group compared to TD and which significantly increased (adjusted p<0.05) at MTT-10wk compared to Baseline group. At MTT-2yr, out of 12, only 1 bacteria (*Rhodovulum sulfidophilum*) significantly decreased (adjusted p<0.05) and there were no change for others (11 out of 12) compared to Baseline.

To understand and confirm the KOs shift after MTT, we explored specific differences in KOs between groups. We identified 5,069 KEGG Orthologs (KOs, functional genes) using HUMAnN2. As shown in Figure 5, comparing ASD at Baseline *vs*. TD, 37 KOs that were significantly different (adjusted p<0.05) (Supplementary Table S3-S5); 22 KOs (out of 37 KOs) were significantly lower (KO Cluster-I), and 15 KOs were higher (KO Cluster-II) (Supplementary Table S6). KO Cluster-I included KOs that encode genes for oxidative stress response, nucleotide-, carbohydrate-, and protein-degradation, and sulfur metabolism (sulfate reduction). At MTT-10wk, the relative abundances of 9 of the 37 KOs significantly increased (adjusted p<0.05) compared to ASD baseline and became similar to TD, and none significantly decreased (Figure 5, Supplementary Table S6). At MTT-2yr there was still a general increase compared to ASD Baseline, but only 3 of the 37 KOs increased significantly compared to Baseline, and none decreased significantly.

KO Cluster-II (Figure 5) consist of 15 KOs including KOs that encode for ion and sugar transporters, carbohydrate degradation and energy production, and terpenoids and polyketides biosynthesis (Supplementary Table S4). These KOs were higher at Baseline compared to TD. At MTT-10wk, one KO significantly decreased, 8 decreased but were not statistically significant, and none significantly increased. At MTT-2yr 12 of the 15 KOs that were higher at Baseline significantly decreased (adjusted p<0.05) and became more similar to TD, and none had significantly increased (Figure 5, Supplementary Table S4). Overall, after MTT (10wk, 2yr), the relative abundance of KOs from Cluster-I and -II became more similar to TD. These findings suggest that MTT had a positive functional impact on children with ASD and it shifted the functional-gene profile of those genes towards the profile of TD children.

For all KOs, longitudinal pairwise comparison between Baseline and MTT-10wks showed 183 KOs were significantly different (adjusted p<0.05); 149 KOs were significantly increased and 34 were decreased after MTT. At MTT-2yrs, 209 KOs were significantly different; 13 KOs were increased but 196 were decreased (Table 1b). Comparison of TD *vs*. MTT (10wks, 2yr) for KOs showed similar trend like ASD Baseline *vs*. MTT (Table S1). So, major changes in the metabolic pathways occurred at MTT-10wks, but additional major and distinct changes occurred at 2 years post-treatment.

Again, considering all KOs, comparing TD *vs*. MTT-10wks, 144 KOs were significantly different (104 KOs were higher and 40 were lower, adjusted p<0.05). For TD *vs*. MTT-2yrs, 58 KOs were significantly different (4 KOs were higher and 54 were lower at MTT-2yrs, adjusted p<0.05) (Table S2-S3). Overall, the KOs became more dissimilar between TD and MTT-10wks (144 different KOs) compared to ASD Baseline (37 different KOs) and remained somewhat more dissimilar at MTT-2yrs (58 different KOs) (Table S2-S3).

We also explored differences in the relative abundances of important functional genes that were not significantly different between Baseline and TD but changed significantly (adjusted p<0.05) after MTT (10wk/2yr). For example, K03190 (ureD, ureH): urease accessory protein (Supplementary Figure S6A), K08717 (utp): Urea transporter (Supplementary Figure S6B), K13990 (FTCD): glutamate formiminotransferase (produce glutamate from histidine) (Supplementary Figure S6C), and K01438 (argE): acetylornithine deacetylase (ornithine and arginine biosynthesis) (Supplementary Figure S6D) were non-significantly higher at Baseline compared to TD but significantly decreased after MTT (10wk, 2yr) (Figure Supplementary S6). One possible reason for a decrease in abundance of these genes and closer to donors after MTT is that major and maintenance donors (median) abundance were also lower than Baseline and TD.

Correlation analysis show links between omics and GSRS

A total of 280 nodes with 420 edges were identified with correlations of R>0.6 or R<-0.6 and adjusted p<0.05. Network analysis was performed only for the ASD Baseline group and did not include fecal metabolites, as no significant changes were observed. The co-occurrence network showed 241 positive (green color) and 179 negative (red) correlations for neurotransmitters/neuroactive molecules, amino acids, indole, taurine, tyramine derivatives, and for sulfur metabolism (Supplementary Figure S7). Metabolite N6-acetyllysine (an amino acid derivative) had the highest 28 negative correlations with KOs and bacteria. However, N6-acetyllysine was neither significantly different between ASD Baseline vs TD, nor changed after MTT. Moreover, metabolites 2-aminophenol sulfate had 18 and 2-oxindole-3-acetate had 19 positive correlations with different bacteria, but these two metabolites were also neither significantly different between ASD Baseline *vs*. TD, nor changed after MTT. Overall, no significant correlation networks were observed for *Prevotella, Bifiobacterium, Desulfovibrio* species and important KOs for oxidative stress, dissimilatory sulfate reduction and other taxa or KOs mentioned in the results section. Among other bacterial species, *Selenomonas sp.-oral-taxon-136* was negatively correlated (R<-0.6, adjusted p<0.05) with four KOs that encode for NAD^+ including K00330 (*nuoA*): and K00340 (*nuoK*): NADH-quinone oxidoreductase subunit A and K, K00346 (*nqrA*) and K00351 (*nqrF*): Na+-transporting NADH: ubiquinone oxidoreductase subunit A and F (Supplementary Figure S8). A KO related to dissimilatory sulfur metabolism, KO K01082 (*BPNT1/cycQ*), which was significantly higher in the Baseline group (Fig 8A), was negatively correlated with *Selenomonas sp.-oral-taxon-136* (Supplementary Figure S9A). *Selenomonas sp.-oral-taxon-136* relative abundance was significantly lower at Baseline *vs.* TD and increased at MTT-10wk (Supplementary Figure S8B). For all participants, KO K01082 showed modest significantly negative correlation with *Desulfovibrio piger* (a sulfur reducer) (Supplementary Figure S9B). We explored the correlation matrix with clinical data (e.g. CARS, GSRS *etc.*) and identified a positive correlation of GSRS with *Nostoc linckia* (R=0.61, *p*=0.006) (Supplementary Figure S7, S10A).

Table S1. Beta Diversity: Jaccard dissimilarity index

A) Bacterial taxonomy

Group 1	Group 2	Permutations	R	p-value	q-value
Baseline	MTT_10wk	999	0.2910	0.001	0.0025
	MTT_2yr	999	0.8225	0.001	0.0025
	TD	999	0.0539	0.066	0.108

B) KOs (KEGG Orthologs)

Group 1	Group 2	Permutations	R	p-value	q-value
Baseline	MTT_10wk	999	0.2605	0.001	0.003
	MTT_2yr	999	0.3292	0.001	0.003
	TD	999	0.0619	0.027	0.040

ANOSIM (ANalysis Of Similarities) was used for the statistical comparison between ASD Baseline and other groups. All p-values are corrected by Benjamin-Hochberg method and assigned as q-values. Overall ANOSIM test values for bacterial distance R=0.45, p=0.001 and for KOs distance R=0.32, p=0.001.

Table S2. Beta Diversity: Bray-Curtis dissimilarity index

A) Bacterial taxonomy

Group 1	Group 2	Permutations	R	p-value	q-value
Baseline	MTT_10wk	999	0.0342	0.135	0.16875
	MTT_2yr	999	0.1259	0.012	0.03
	TD	999	0.0516	0.077	0.1155

B) KOs (KEGG Orthologs)

Group 1	Group 2	Permutations	R	p-value	q-value
Baseline	MTT_10wk	999	0.1275	0.004	0.0128
	MTT_2yr	999	0.1555	0.001	0. 0128
	TD	999	0.0402	0.112	0.1360

ANOSIM (ANalysis Of Similarities) was used for the statistical comparison between ASD Baseline and other groups. All p-values are corrected by Benjamin-Hochberg method and assigned as q-values. Overall ANOSIM test values for bacterial distance R=0.14, p=0.001 and for KOs distance R=0.16, p=0.001.

Table S3. Taxonomy and KOs comparison between groups

A) Bacterial taxonomy comparisons (all 5,272 taxa)

	ASD at Baseline *vs.*		
ASD at Baseline *vs.* TD	MTT-10wks	MTT-2yrs	differences
0	650	12	Higher/increased compared to Baseline
371	16	1599	Lower/decreased compared to Baseline
371	666	1611	Total

**ASD at Baseline *vs.*** (out of 371 taxa different between Baseline ASD *vs.* TD)		
MTT-10wks	MTT-2yrs	differences
97	0	Increased compared to ASD Baseline
1	60	Decreased compared to ASD Baseline
98	60	Total

B) KOs (KEGG Orthologs) comparisons (all 5,069 KOs)

	ASD at Baseline *vs.*		
ASD at Baseline *vs.* TD	MTT-10wks	MTT-2yrs	differences
15	149	13	Higher/increased compared to Baseline
22	34	196	Lower/decreased compared to Baseline
37	183	209	Total

**ASD at Baseline *vs.*** (out of Baseline *vs.* TD = 37KOs)		
MTT-10wks	MTT-2yrs	differences
9	3	Increased compared to ASD Baseline
01	12	Decreased compared to ASD Baseline
10	15	Total

Univariate comparison of the relative abundance (after $\log_{10}$ transformation) of bacterial species (n=5,272) and KEGG Orthologs (KOs, n=5,069) between groups. All statistically significant numbers are with adjusted $p<0.05$.

Table S4. Differences in number of taxa between groups

**ASD at Baseline *vs.* MTT-2yrs (12 significantly increased taxa at MTT-2yrs**	
Halocynthiibacter_arcticus	*Alteromonas_australica*
Bacteroides_intestinalis	*Pseudomonas_syringae_group_genomosp.3*
Butyricimonas_faecalis	*Pseudomonas_tolaasii*
Actinomyces_pacaensis	*Xanthomonas_euvesicatoria*
Dehalobacter_restrictus	*Streptomyces_lividans*
Candidatus_Erwinia_haradaeae	*Blautia_coccoides*

**ASD at Baseline *vs.* MTT-10wks (16 significantly decreased taxa at MTT-10wks)**	
Pseudomonas_sp._GR_6-02	*Methylobacterium_oryzae*
Pseudoalteromonas_issachenkonii	*Acetobacter_sp._B6*
Mycobacterium_sp._PYR15	*Sphingorhabdus_sp._M41*
Bacteroides_xylanisolvens	*Pusillimonas_sp._ye3*
Alistipes_finegoldii	*Enterobacter_cloacae_complex_sp._FDA-CDC-AR_0132*
Synechococcus_sp._PCC_11901	*Leclercia_sp._119287*
Serratia_ficaria	*Xanthomonas_perforans*
Acinetobacter_baylyi	
Stenotrophomonas_sp._ASS1	

**TD *vs.* MTT-2yrs (6 significantly higher taxa at MTT-2yrs)**	
Bacteroides_intestinalis	*Candidatus_Erwinia_haradaeae*
Butyricimonas_faecalis	*Pseudomonas_syringae_group_genomosp.3*
Vibrio_sp._EJY3	*Xanthomonas_euvesicatoria*

Table S5. Taxonomy and KOs comparison between TD and all ASD groups

A) Bacterial taxonomy comparisons

vs. TD (comparing all taxa)			
ASD Baseline	MTT-10wks	MTT-2yrs	Differences
0	48	6	Higher in ASD *vs* TD
371	387	2984	Lower in ASD *vs* TD
371	435	2990	Total

B) KOs (KEGG Orthologs) comparisons

vs. TD (comparing all KOs)			
ASD Baseline	MTT-10wks	MTT-2yrs	Differences
15	104	4	Higher in ASD *vs* TD
22	40	54	Lower in ASD *vs* TD
37	144	58	Total

Univariate comparison of the relative abundance (after $\log_{10}$ transformation) of bacterial species (n=5,272) and KEGG Orthologs (KOs, n=5,069) between groups. All statistically significant numbers are with adjusted $p<0.05$.

Table S6. List of significantly different 37 KOs/metabolic pathways in ASD Baseline *vs.* TD children from Figure 5.

**KO Cluster-I, significantly lower KOs in ASD Baseline**						
No.	Super Pathways	Sub-pathways	KOs	Gene	In ASD	*P*-values
1	Carbohydrate metabolism	Starch and sucrose metabolism	K05343	treS	**Lower ↓**	***
		Galactose metabolism	K08302	gatY-kbaY		***
2	Glycan biosynthesis and metabolism	Peptidoglycan biosynthesis	K07009	gatD		***
3	Nucleotide/cofactors and vitamins Metabolism	Pyrimidine metabolism/ One carbon pool by folate	K03465	thyX, thy1		*
		Pyrimidine metabolism	K06966	ppnN		***
4	Protein families: metabolism	Peptidoglycan biosynthesis and degradation proteins	K08309	slt		***
5	Signaling and cellular processes	Cell growth	K06400	spoIVCA		***
		Transporters	K16211	malY,malT		*
			K16926	htsT		*
6	Environmental Information Processing	Signal transduction	K04771	degP, htrA		***
		Membrane transport	K10112	msmX, sugC, msiK		*
7	Genetic information processing	Chromosome and associated proteins	K02499	yabN		***
		Bacterial secretion system	K03074	secF		***
		Transcription factors	K03556	malT		***
		Replication and repair	K03702	uvrB		*
		Transfer RNA biogenesis	K04094	trmFO, gid		*
		Replication and repair	K06919	NA		*
			K07481	NA		***
8	Unclassified: metabolism	Unclassified	K05919	dfx		**
			K05985	rnmV		***
			K07099	NA		***
			K10254	ohyA, sph		*
**KO Cluster-II significantly higher KOs in ASD Baseline**						
1	Carbohydrate metabolism	Glycolysis / Gluconeogenesis/TCA cycle	K00174	korA, oorA, oforA	**Higher ↑**	*
			K00382	DLD, lpd, pdhD		***
		Pentose and glucuronate interconversions	K00963	UGP2, galU, galF		***
2	Glycan biosynthesis and metabolism	N-Glycan biosynthesis	K00721	DPM1		***
3	Metabolism of terpenoids and polyketides	Terpenoid backbone biosynthesis	K02523	ispB		***
4	Energy metabolism	Oxidative phosphorylation	K00330	nuoA		***
			K00340	nuoK		*
		Sulfur metabolism	K01082	cysQ, BPNT1		***
5	Signaling and cellular processes	Transporters	K03313	nhaA		***
			K08169	yebQ		***
			K11537	xapB		***
6	Unclassified	Unclassified	K00346	nqrA		*
			K00351	nqrF		***
			K00986	ltrA		***
			K07164	NA		***

Single asterisk* indicates p <0.05, *triple * asterisks* indicate p <0.001, NA-gene name not available. All p-values are FDR corrected.

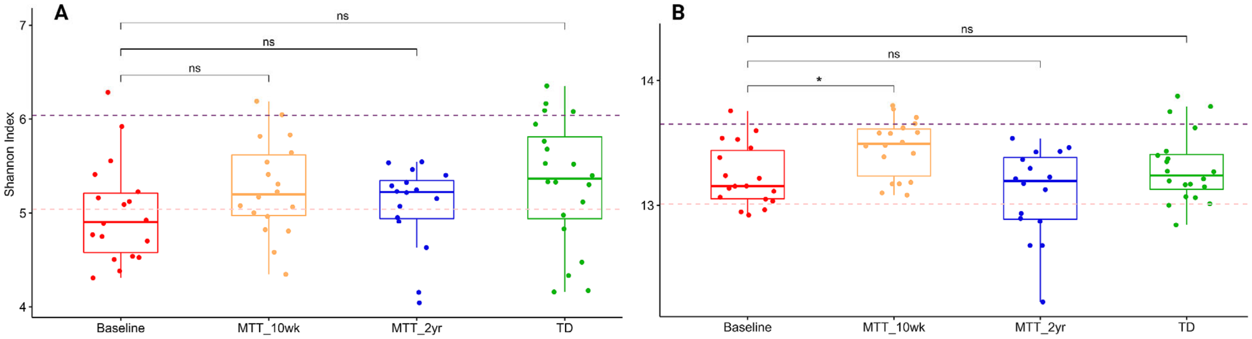

Figure S1. The alpha-diversity before and after MTT in children with ASD. The Shannon diversity index of (A) bacteria and (B) KEGG Orthologs between groups. Each colored dot represents a study participant. Purple dashed lines represent the mean of maintenance (n=2) and pink, the median of major donors (n=5). TD: Typically Developing. * Asterisk represent significant differences between ASD Baseline, and the other groups (*p <0.05, ns not significant, all p-values are BH method FDR corrected).

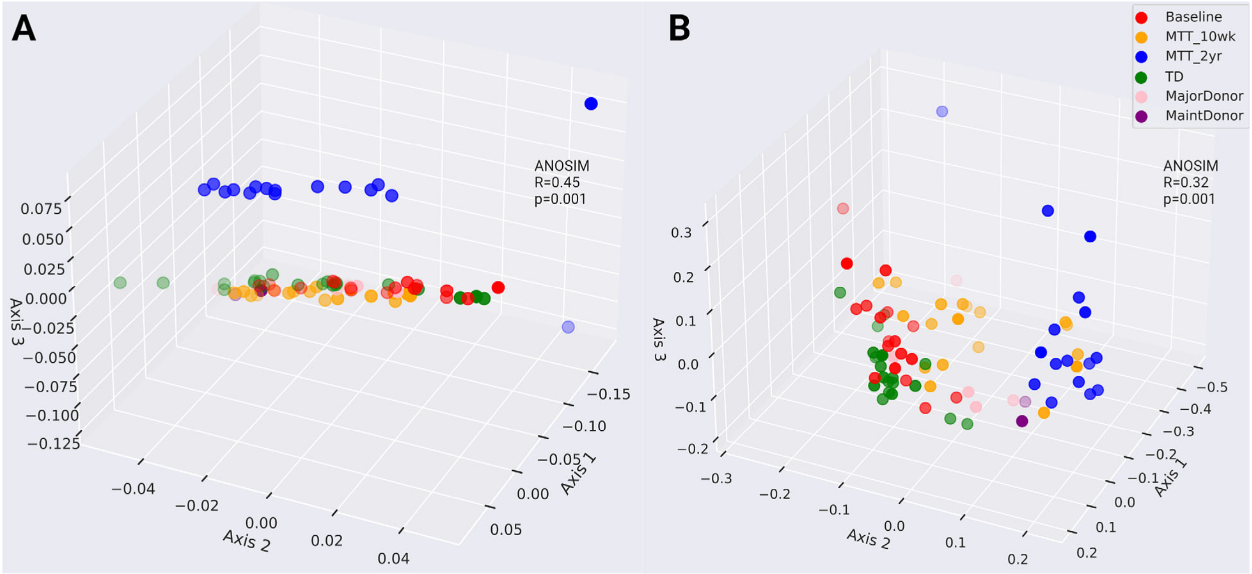

Figure S2. Jaccard distance dissimilarity index before and after MTT in children with ASD. (A) bacterial community and (B) KEGG Orthologs between groups. Each colored dot represents a study participant. TD: Typically Developing, Main Donor: Maintenance donor, ANOSIM: Analysis of similarities.

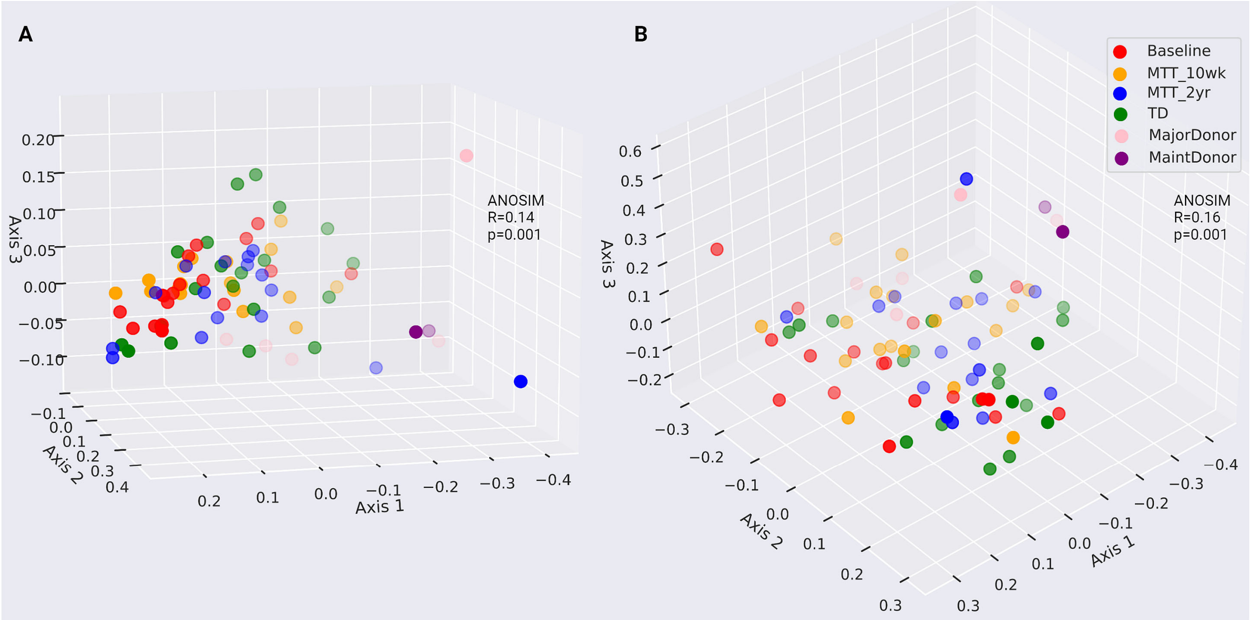

Figure S3. The beta-diversity before and after MTT in children with ASD. The Bray-Curtis distance dissimilarity index of (A) bacteria and (B) KEGG Orthologs between groups. Each colored dot represents an ASD individual. ASD: Autism Spectrum Disorders, TD: Typically Developing, MaintDonor: Maintenance donor. ANOSIM (Analysis of similarities) was used for the statistical comparison between ASD Baseline and other groups (see more details Table S4).

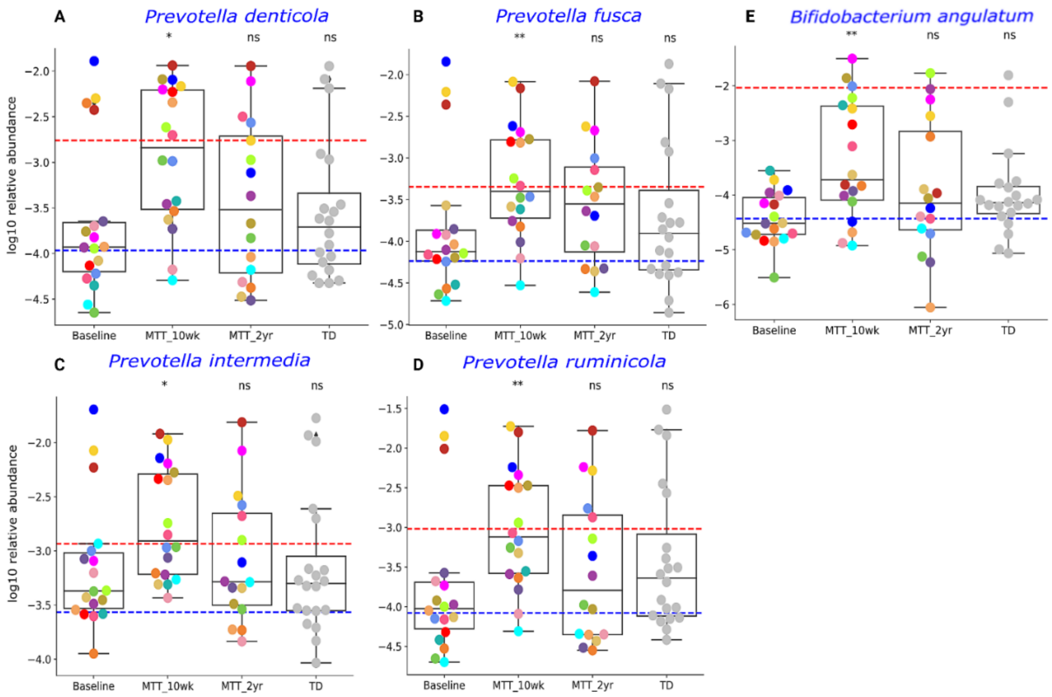

Figure S4. Univariate comparison of the relative abundance (after $\log_{10}$ transformation) of different taxa. *Prevotella* species A) *P. denticola*, B) *P. fusca*, C) *P. intermedia*, D) *P. ruminicola* and E) *Bifodobacterium angulatum* of ASD Baseline *vs*. MTT (10wk, 2yr) and TD. Red dashed lines represent the mean of maintenance (n=2) and blue for median of major donors (n=5). Each colored dot represents one ASD individual and grey colored dots represent TD. Asterisks represent significant differences between ASD Baseline, and the other groups (*Single asterisk indicates p<0.05, **double asterisks indicate p<0.01, ns not significant, all p-values are FDR corrected).

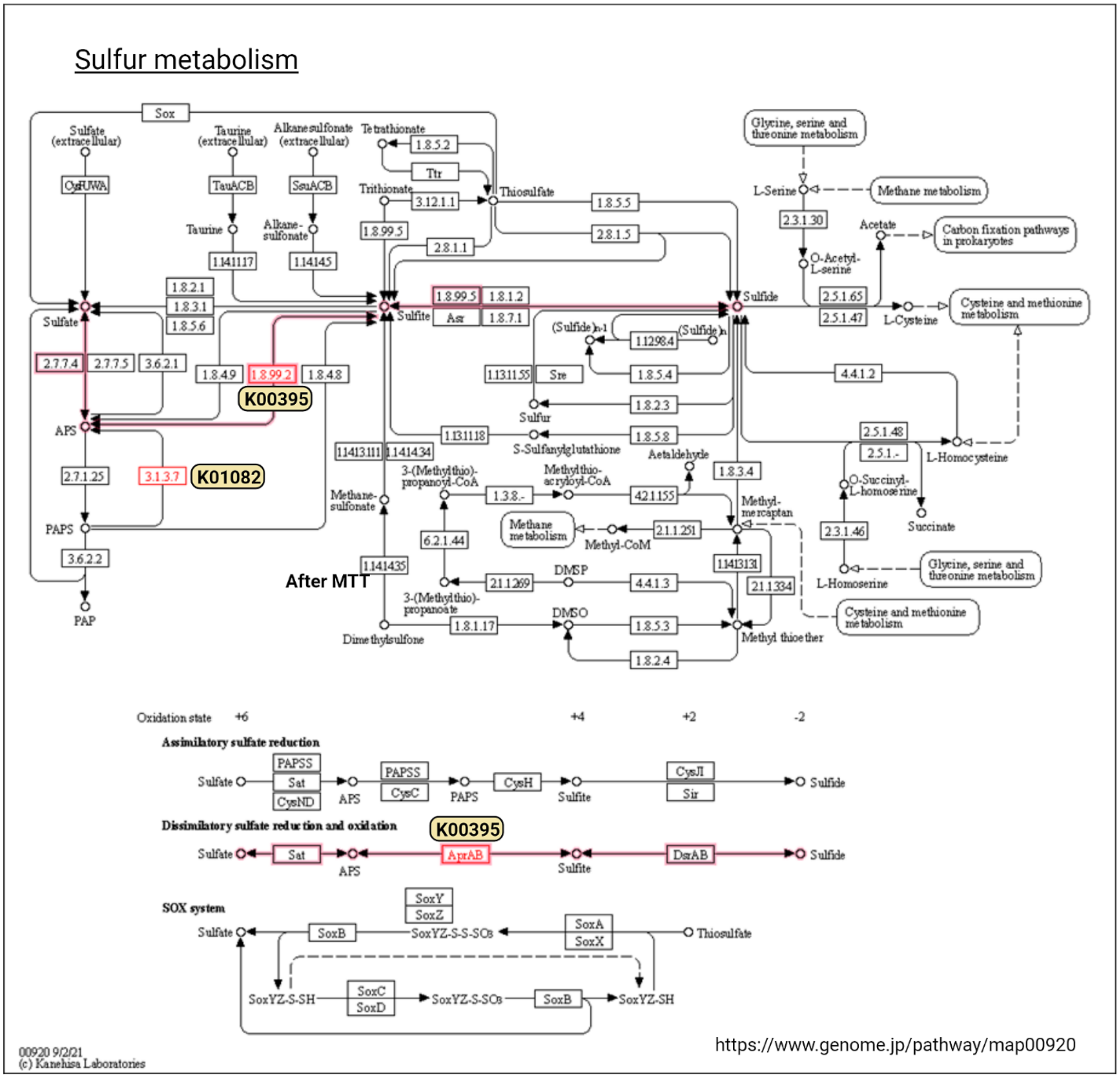

Figure S5. Overview of sulfur metabolism. Yellow colored KO K01082 converts PAPS to APS and KO K00395 converts APS to sulfite in dissimilatory sulfate reduction (Figure 10). Figure generated from KEGG database website https://www.genome.jp/pathway/map00920.

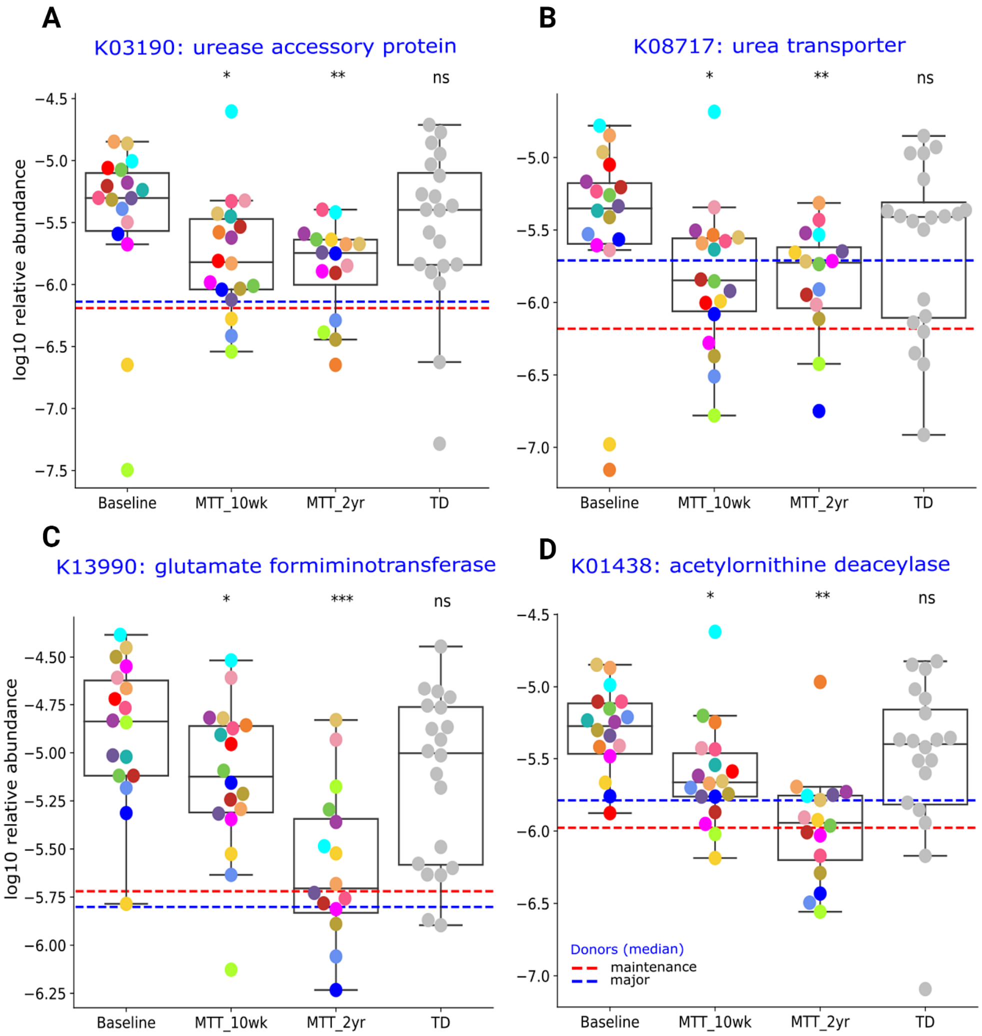

Figure S6. Univariate comparison of the relative abundance (after $\log_{10}$ transformation) of gut microbiome genes/KOs that changed significantly after MTT in ASD but did not become similar to TD. A) K03190: urease accessory protein, B) K08717: Urea transporter, C) K13990: glutamate formiminotransferase, D) K01438: acetylornithine deacetylase. Dashed lines represent the median of donors. Red dashed lines represent the mean of maintenance (n=2) and blue the median of major donors (n=5). Colored dots represent ASD individuals and grey colored ones refer to TD. Asterisks represent significant differences between ASD Baseline, and the other groups (*Single asterisk indicates $p < 0.05$, **double asterisks indicate $p < 0.01$, triple *** asterisks indicate $p < 0.001$, ns not significant, all p-values are FDR corrected). ASD: Autism Spectrum Disorders, TD: Typically Developing.

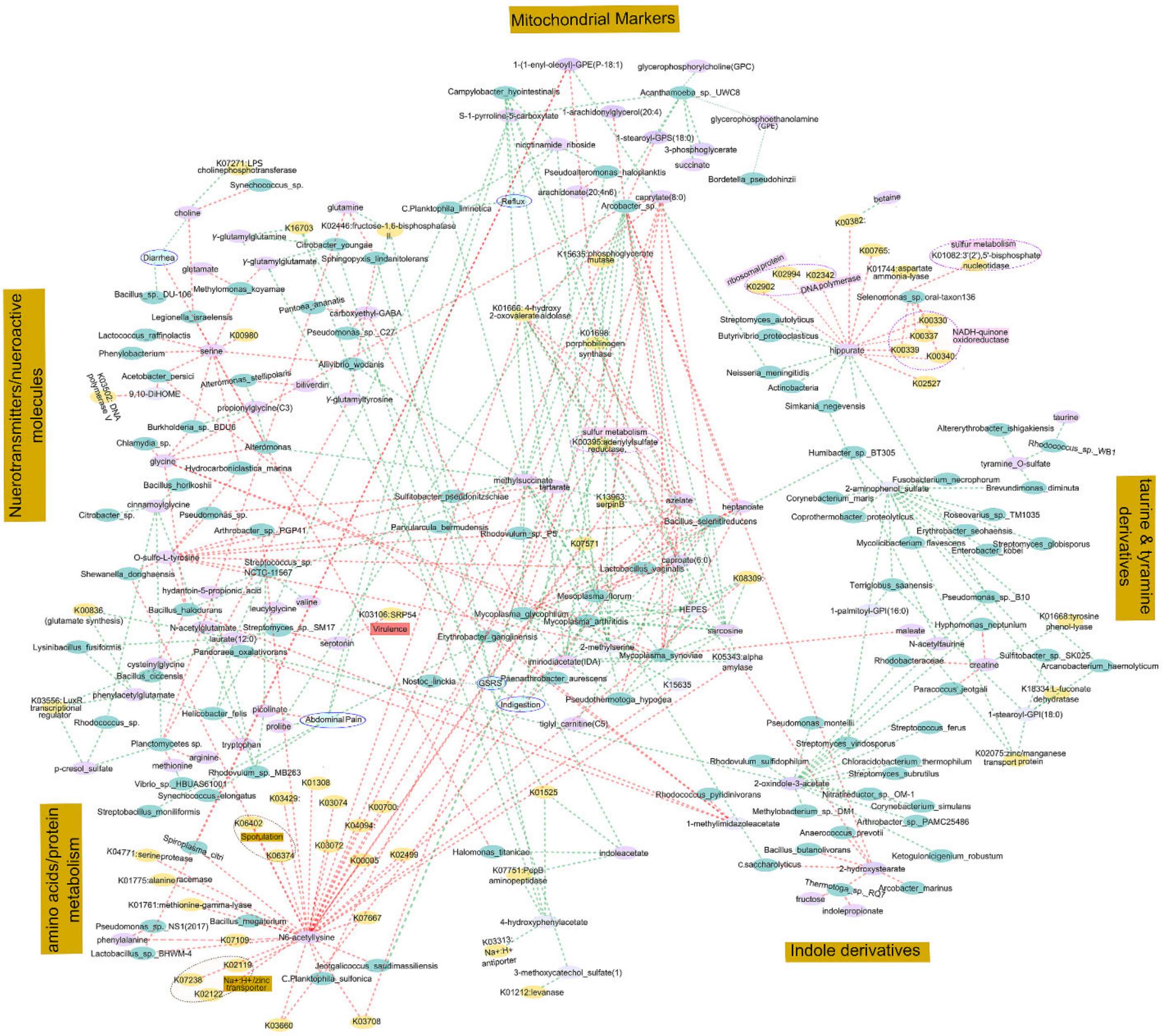

Figure S7. Network analyses of microbes, microbial metabolic pathways, plasma metabolites and ASD symptoms for children with ASD at Baseline. Green dotted lines are positive and red dotted lines are negative correlations. All p-values are adjusted (p<0.05), and correlation values are R>0.6 or R<-0.6.

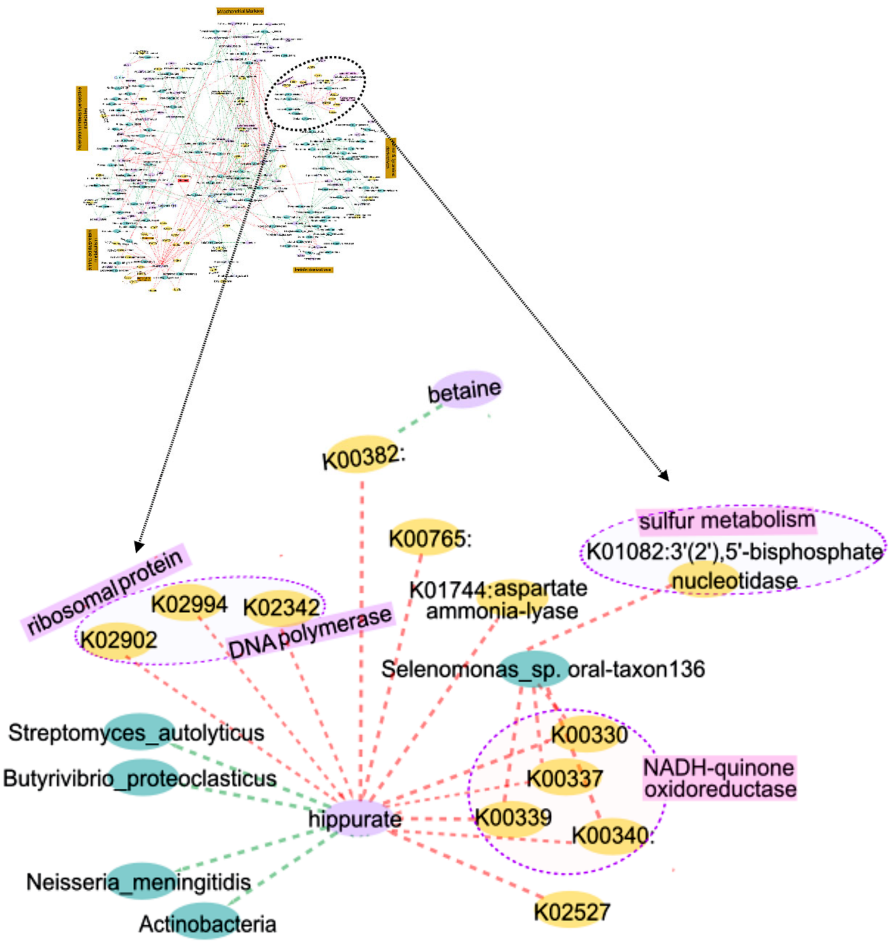

Figure S8. Subset of correlation network between microbiome and pathways with plasma metabolites. *Selenomonas sp.-oral-taxon-136* negatively correlated ($R<-0.6$, adjusted $p<0.05$) with NAD+ producing KOs K00330, K00337, K00339, K00340 and dissimilatory sulfur metabolism associated KO K01082. Green lines represent positive and red lines negative correlations. The full network is shown in Figure S6.

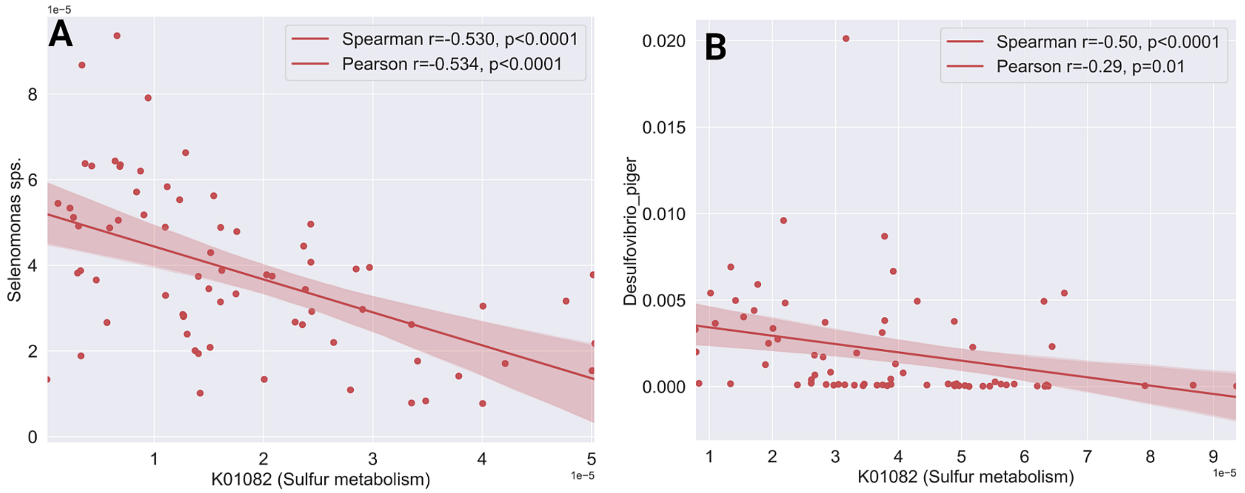

Figure S9. Correlation tests between taxa and microbial genes. A) between relative abundance of K01082:3′(2′),5′-biphosphate nucleotidase and *Selenomonas* species, B) between relative abundance of K01082:3′(2′),5′-biphosphate nucleotidase and *Desulfovibrio piger*.

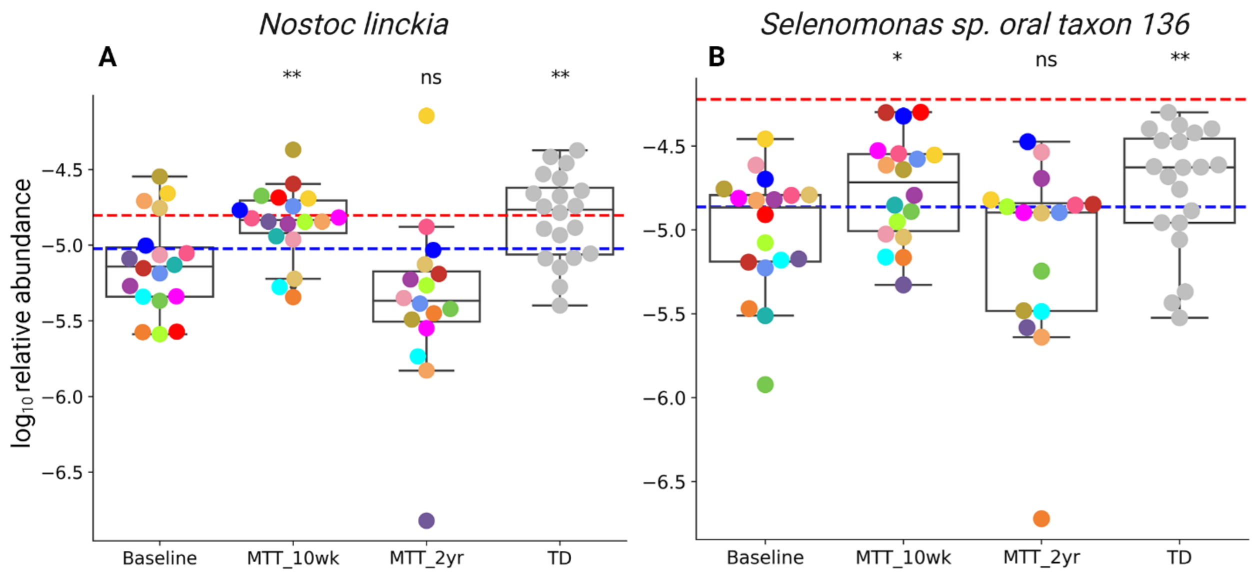

Figure S10. Univariate comparison of the relative abundance (after $\log_{10}$ transformation) of different taxa. A) *Nostoc linckia* and B) *Selenomonas sp. oral taxon-136* species before and after MTT. Red lines for maintenance (n=2) and blue for major (n=5) donors. Each colored dot represents one ASD individual and grey colored dots represent TD (typically developing). Asterisks represent significant differences between ASD Baseline, and the other groups (*Single asterisk indicates p<0.05, **double asterisks indicate p<0.01, ns not significant, all p-values are FDR corrected).

References:

1. Kang, D.-W.; Adams, J.B.; Vargason, T.; Santiago, M.; Hahn, J.; Krajmalnik-Brown, R. Distinct Fecal and Plasma Metabolites in Children with Autism Spectrum Disorders and Their Modulation after Microbiota Transfer Therapy. *mSphere* **2020**, *5*, doi:10.1128/MSPHERE.00314-20.
